# Achieving clinically meaningful outcomes in digital health: a six-step, cyclical precision engagement framework (ENGAGE)

**DOI:** 10.3389/fdgth.2025.1713334

**Published:** 2026-01-13

**Authors:** Anne-Kathrin Eiselt, Suzanne Kirkendall, Engelina Xiong, David Langner, Micah Goldfarb

**Affiliations:** 1 Professional Psychology Department, George Washington University, Washington, DC, United States; 2 Ipsos, Behavioral Science Center, New York City, NY, United States; 3 BVA Nudge Consulting, North America, New York City, NY, United States

**Keywords:** digital health intervention (DHI), user engagement, behavior change, personalization, precision-health, Just-in-Time Adaptive Interventions (JITAI), real-world evidence

## Abstract

By leveraging everyday technologies such as mobile apps, wearables, and AI-enabled tools, digital health interventions (DHIs) offer new pathways to integrate self-management and intervention programs into the fabric of daily life, while bridging gaps in care through continuous, context-aware support. Yet many tools underperform clinically because digital engagement (“screen time”) is conflated with impact, while behavioral science is retrofitted, if applied at all. We propose the ENGAGE Framework: a cyclical, six-step model of precision engagement that integrates user needs, behavioral science, and adaptive personalization to transform initial curiosity into sustained real-world habits. By leveraging available data, users can be segmented according to their need (Step 1: Enroll & Segment), targeted with the most relevant and engaging message to increase micro-engagement (Step 2: Nudge & Hook), and persuaded to engage in real-world health behavior change (Step 3: Guide Behavior). From this macro-engagement step, additional core behavioral science principles are used to reinforce the real-world behaviors long enough to positively impact health outcomes (Step 4: Anchor Habits), while measuring progress (Step 5: Generate Evidence) to inform adaptive and optimized engagement strategies (Step 6: Expand & Evolve with AI) for tailored interventions and communications based on user characteristics, context, and clinical data for both new and existing users. Each step of the ENGAGE Framework maps to evidence-based techniques, implementation tactics (e.g., integration pathways and operational deployment strategies), and metrics that help translate superficial engagement into long-lasting behavior change and measurable clinical outcomes. We synthesize relevant engagement literature, identify gaps and challenges (e.g., measurement heterogeneity, lack of focus on macro-engagement, product development challenges, ecosystem barriers), and offer a practical checklist for innovators. By focusing on who needs what support, when and why, ENGAGE aims to help DHI developers and researchers design interventions that are effective, equitable, and empirically testable.

## 1 Introduction

Digital health interventions (DHIs) are technology-based tools, platforms, or programs that deliver health services, support, or behavior change through digital means such as mobile apps, wearable devices, web platforms, telehealth, or AI-driven systems. They are transforming healthcare by enhancing access, equity, empowerment, operational efficiency, and cost-effectiveness, while also improving clinical outcomes (1–3). For patients, remote patient monitoring (RPM) and telehealth extend healthcare services beyond geographical limitations, making them more accessible and equitable (4–7). Conversational agents and AI-guided triage processes can reduce organizational resources and individual cognitive load, and support guided self-management and informed clinical decision-making, which enhances patient engagement and empowerment (8, 9) leading to improved patient priority outcomes (10–12).

For health systems and products, these tools hold tremendous potential to enhance societal well-being by improving the viability, proficiency, transparency, and personalization of healthcare services. They also strengthen the healthcare system through faster information exchange, greater patient-centricity, reduced provider burnout, resource optimization, reimbursement advantages, individualized recommendations, support for measurement- and value-based care, improved diagnostics, and long-term cost reduction through early detection, prevention, and improved outcomes (9, 13–15).

However, despite the promise and rapid rise of DHIs, most applications (“apps”) lack convincing long-term clinical impact, leaving healthcare systems, payers, and insurers skeptical about their potential for sustained benefits (16–19). Systematic reviews show inconsistent, or even absent, long-term clinical benefit, largely attributable to shallow or declining user engagement (20–29).

Many digital health innovators mistakenly equate engagement with impact, overlooking that interaction with a DHI on its own is rarely sufficient to drive lasting health outcomes. Without a structured approach to move users beyond enrollment and in-app interactions toward real-world behavior change, most DHIs will struggle to convert initial motivation into measurable and sustained clinical benefits (19, 30, 31).

Applied behavioral science provides the knowledge and tools to bridge this *engagement-impact gap*, yet integration into product lifecycles remains sporadic (32). To address this gap, we outline a unifying, pragmatic framework (ENGAGE) to guide the design, evaluation, and iteration of DHIs targeting chronic conditions and long-term care, as well as wellness, prevention, and health maintenance.

The ENGAGE Framework, a cyclical, six-step model for precision engagement in digital health interventions, integrates behavioral science, real-world measurement, and adaptive personalization to convert initial curiosity into sustained real-world behavior change. By uniting recruitment, micro-engagement (digital interactions), macro-engagement (real-world behavior change), habit formation, outcome evaluation, and AI/ML-driven iterations within a single operational blueprint, it offers a scalable approach to achieve meaningful and measurable health impact.

In this manuscript, we focus on chronic condition and health management, which refers to long-term health conditions such as cardiovascular disease, diabetes, obesity, chronic respiratory diseases, and mental health conditions like depression and anxiety, which require ongoing management, behavior change, and sustained engagement to prevent worsening, improve quality of life, and achieve patient priority health goals (WHO, 2020). While some principles described here may apply to regulated digital therapeutics (DTx) and prescription digital therapeutics (PDTs), our focus is on digital health interventions more broadly, including both wellness and clinical tools, and not specifically on the formal regulatory approval process required for medical devices.

The objective of this article is to (a) consolidate multidisciplinary evidence on digital engagement, behavior change, and personalization, leading to a new viewpoint, (b) introduce the ENGAGE framework, detailing theory-informed techniques, example tools, and metrics for each of the six steps, (c) provide an actionable implementation checklist for innovators and product developers to integrate foundational behavioral science principles into applications, and (d) highlight research and reporting gaps, as well as challenges to guide future research efforts.

### 1.1 Positioning the ENGAGE framework within the existing literature

Several conceptual models and taxonomies have been proposed to guide engagement design in digital health interventions (DHIs), including the *Person-Based Approach* (33), the *IDEAS Framework* (34), the *Effective Engagement Framework* (31), and the *AIM-ACT* Framework (35). These models have advanced the field by emphasizing user-centered design, iterative development, and the importance of linking engagement to behavioral theory. However, most existing frameworks focus either on early intervention design phases, within-intervention adaptation, or measurement taxonomies in isolation. Few explicitly address the cyclical, closed-loop nature of engagement optimization that bridges initial user acquisition, micro-engagement, macro-engagement, habit formation, and adaptive iteration at scale.

For example, the Person-Based Approach (33, 36) provides detailed qualitative methods to elicit user needs during development but offers less guidance on sustaining engagement beyond deployment or integrating behavioral data for real-time adaptation. The IDEAS Framework (34) offers a stepwise process from ideation to evaluation but is primarily oriented toward developing workflows rather than operationalizing ongoing personalization. Similar process frameworks (16) address common challenges for engagement by focusing on user-centered designs but lack the behavioral science foundation and personalization components. The Effective Engagement Framework (31) usefully distinguishes between micro-engagement (“little e”) and macro-engagement (“big E”), yet does not prescribe mechanisms to systematically transition users from digital interactions to sustained real-world health behaviors.

While the AIM-ACT Framework (35) shares the same conceptual ideas, it operates at different levels of scope, purpose, and implementation detail. The AIM-ACT Framework offers a readiness-state model for tailoring Just-in-Time Adaptive Interventions (JITAIs), guiding intervention delivery across sequential states of awareness, interest, motivation, action, continuation, and tapering. While highly valuable for optimizing within-intervention decision-making, AIM-ACT is narrower in scope, focusing on micro-level adaptation once a user is already engaged. Unlike design methodologies such as person-based or human-centered approaches, and unlike frameworks such as IDEAS or AIM-ACT, ENGAGE provides an end-to-end, mechanism-focused engagement cycle that links behavioral drivers with real-world outcomes and adaptive personalization (37, 38).

The ENGAGE model is intended to complement, not replace, established hybrid effectiveness–implementation frameworks such as RE-AIM (39, 40) and PRISM (41), which provide structured approaches for evaluating reach, adoption, implementation fidelity, and sustainability. While ENGAGE focuses specifically on the mechanisms of digital engagement and the behavioral and contextual drivers that shape user participation, frameworks like RE-AIM and PRISM offer broader evaluation scaffolds that can guide real-world assessment and scale-up. Together, these models enable a more complete understanding of both how digital health interventions work and under what implementation conditions they are most likely to succeed.

Other useful approaches to standardize taxonomies and reporting protocols are *COM-B* and *BCTTv1* (42, 43), which help identify barriers as well as the “active ingredients” for behavior change and mapping them according to their impact domain (i.e., capability, opportunity, motivation). Other frameworks like the *Intervention Mapping (IM) taxonomy* (44), the conceptual framework for engagement measurement (45), the *5-Element Engagement Reporting* Framework (46), and the Engagement Mapping Framework (47), all contribute valuable clarity in specifying engagement targets, pathways and steps, measures, and doses, as well as debiasing strategies. They also call for more consistency and transparency on what type of engagement is being targeted (e.g., emotional, cognitive, behavioral), how engagement is elicited (e.g., gamification, reminders, coaching), how it is defined and measured (e.g., app analytics, alliance, usability), and what dose of engagement is required for change (48). However, they do not provide a comprehensive operational blueprint that integrates these into intervention design, delivery, and adaptation.

In contrast to these current models, the ENGAGE Framework addresses the full engagement lifecycle, from initial recruitment and segmentation to micro- and macro-engagement, habit formation, outcome measurement, and AI-driven iteration. Although grounded in behavioral science, ENGAGE harmonizes evidence from UX/engineering, data science, and clinical research by providing a shared mechanism-focused structure that links user behavior, digital design decisions, and measurable clinical outcomes. It also embeds measurement as a dedicated step and treats personalization as dual-purpose, enhancing both the targeting of new users and the optimization of interventions for existing users. In this way, ENGAGE complements the above frameworks, by providing a broader operational architecture and unified stepwise process within which readiness-based adaptation can be deployed at scale.

The ENGAGE framework is motivated in part by the limitations of small-sample, short-term DHI studies and the need for scalable, mechanism-based models that synthesize behavioral, clinical, and digital metrics through real-world data and emerging AI/ML tools. It provides three key innovations over existing approaches. First, it explicitly links micro- and macro-engagement within a unified process, ensuring that digital interactions (micro-engagement) are designed as conduits to real-world behavior change (macro-engagement), rather than treating them as separate or implied outcomes. Second, it integrates behavioral science principles at every stage, from initial segmentation and outreach to digital activation and habit anchoring, mapping each step to theory-informed techniques, measurement strategies, and implementation tactics. This avoids the common pitfall of retrofitting behavioral science *post hoc* or applying it selectively to certain features. Third, ENGAGE incorporates a closed-loop, AI/ML-enabled optimization cycle (Steps 5 and 6) in which outcome and engagement data continuously refine both the recruitment of new users and the personalization of interventions for existing users. This dual focus is rarely present in prior frameworks, which typically address either recruitment or retention, but not both as part of the same adaptive process. The result is a cyclical, precision engagement architecture that moves beyond static or linear models.

By uniting evidence-based behavioral techniques, real-world measurement, and adaptive personalization within a single operational blueprint, ENGAGE offers a pragmatic, scalable, and testable model for converting initial patient curiosity into sustained health impact. It differs from prior frameworks not by replacing them, but by integrating their strengths into an end-to-end system designed for implementation in continuously evolving digital health ecosystems and varying personal needs and contexts.

## 2 The engagement-impact gap: behavioral and design pitfalls

The above-mentioned frameworks, as well as ENGAGE, were developed with the same goal of combating the numerous challenges that can hinder the real-world adoption and integration of digital health tools. While data privacy, interoperability, ethical and equity concerns, regulatory and quality oversight, and the need for multidisciplinary collaboration and change management are prominent barriers (49, 50), one of the biggest challenges to achieving clinical impact and effectiveness is sustained user engagement (24, 25, 51–55).

Digital implementation science continues to report striking attrition curves: the median 90-day retention across commercially available health apps is less than 10% (22) and the dose-response relationship between “time-on-app” and clinical change is weak (48). Assessments of adherence are extremely inconsistent and show as low as 6% average adherence rates after 30 days (46), with generally lower adherence in real-world use cases compared to research studies (22, 56). There is evidence that randomized controlled trials (RCTs) routinely achieve significantly higher retention than real-world deployments, illustrating a persistent “efficacy–effectiveness gap” in real world settings (20, 57, 58). Adherence is often inflated through trial incentives, intensive onboarding, using structured and simplified versions of the intervention, and selective recruitment using strict inclusion and exclusion criteria (often recruiting highly motivated and often healthier populations). These factors are rarely feasible at scale in a complex environment with multiple competing priorities (28, 59). These adherence struggles likely explain the lack of convincing long-term clinical impact of commercially available DHIs. Without sufficient exposure to behavior change interventions and implementation of effective strategies that target real-world and meaningful behavior changes, DHIs will continue to fall short of their promise.

This problem is not new. Patient and user engagement through DHIs has been the focus of extensive research over the past decade, highlighting its unique position to deliver scalable, cost-effective, and accessible health support; solving engagement barriers is critical because modest improvements can yield outsized benefits in public health, equity, and system efficiency. Below we synthesize the current evidence about why digital engagement so often stalls.

### 2.1 Defining engagement with digital health interventions

Engagement is a central construct linking digital intervention usage to downstream clinical outcomes, yet its conceptualization and operationalization remain highly inconsistent across the literature and consumer-facing products. This lack of standardization hampers the ability to draw valid conclusions, compare interventions, or develop scalable, evidence-based algorithms to enhance engagement based on concrete insights about what works in real-world settings (52, 60, 61).

While commonly measured through usage metrics such as downloading, log-ins, feature clicks, or time spent within an app, engagement of this sort cannot be equated with adherence and clinical impact (21, 62). Instead, it should be understood as a process with multiple layers, requiring both interaction with the digital product at the intended frequency and duration, as well as meaningful application and generalization in everyday life (35, 46, 63).

A helpful taxonomy proposed by Cole-Lewis et al. (31) presents a two-tier distinction between micro-engagement and macro-engagement, also referred to as “little e” and “big E” engagement, respectively. Micro-engagement (“little e”) refers to the observable interactions with DHIs themselves. This includes behaviors such as signing up, logging in, tapping or swiping through content, responding to prompts or nudges, or interacting with features like trackers or chatbots. These behaviors reflect the user's exposure to, and interaction with the intervention and are essential for delivering the core intervention components of the program. However, higher micro-engagement alone does not necessarily translate into clinical or behavioral change. Macro-engagement (“big E”), in contrast, refers to the adoption of real-world health behaviors that the intervention is designed to support, such as increased physical activity, medication adherence, improved sleep routines, dietary changes, or therapy compliance. It represents the externalization of the intended behavioral outcomes and is ultimately what drives clinical improvement.

For DHIs to be effective in providing sustained clinical benefits, micro-engagement must serve as a causal bridge to macro-engagement. The strength of this bridge depends on the quality of the intervention's behavioral design, including the integration of evidence-based techniques, personalized prompts, and feedback loops, and should be evaluated using systematic, theory-driven approaches (31, 52, 63, 64). Creating a sturdy bridge between micro- and macro-engagement is a foundational component of the ENGAGE Framework (Steps 2 and 3).

### 2.2 Multi-level determinants of engagement

As described before (31), user engagement with DHIs, both on the micro and macro level, is a critical determinant of their real-world effectiveness. Yet, despite increasing adoption of digital health tools, sustained engagement remains a persistent challenge. A growing body of literature underscores that engagement is shaped by a complex constellation of factors, ranging from user-specific characteristics to program design and technology interfaces (24, 31, 52, 56, 64).

A comprehensive categorization by Borghouts et al. (52) delineates these determinants into three interrelated domains: (a) user-related factors, (b) program-related factors, and (c) technology- and context-related factors. Each domain encompasses a spectrum of psychological, behavioral, and environmental influences on engagement (see Table 1).

**Table 1:** Multi-level determinants of engagement.

Domain	Key factor	Insight/Impact	Example references
User-Related Factors	Demographics	Explains ∼15% variance of usage; women engage more; older users face literacy barriers.	(22, 52)
	Clinical Profile	Severe symptoms predict early dropouts, which apply across mental health and chronic care.	(133, 138)
	Personality & Motivation	Conscientiousness and self-efficacy predict use; low intrinsic motivation is linked to attrition.	(63, 234)
	Beliefs & Attitudes	Tech beliefs, illness perceptions, and self-efficacy shape initial and sustained engagement.	(235, 236)
	Digital Literacy & Experience	Low digital literacy and lack of experience hinder uptake, especially in underserved populations.	(118, 237)
	Daily Life Integration	Difficulty integrating tools into routines is a key barrier to sustained engagement.	(238)
Program-Related Factors	Content Quality	Multimedia/interactive content helps; long or non-credible content reduces engagement.	(64, 149)
	Cultural Fit & Relevance	Alignment with user values and health beliefs increases relevance and adherence.	(239, 240)
	Perceived Usefulness	Perceived value and benefit drive ongoing use; lack of utility leads to attrition.	(63, 241)
	Guidance & Support	Human support yields higher engagement; supported models outperform fully digital.	(76, 143, 242)
	Social Connectedness	Peer interaction boosts adherence via motivation, accountability, and relatedness.	(82, 142)
	Perceived Impact	Tangible improvements reinforce continued use through positive feedback loops.	(90)
Technology & Context Factors	Usability & Design	A user's experience is directly shaped by product design and usability; poor experiences increase the risk of abandonment. A user-centric, iterative design approach can prevent disengagement.	(56, 243)
	Technical Issues	Bugs, crashes, or poor compatibility reduce use, especially for less tech-savvy users.	(85)
	Cost & Access	High cost or device/internet requirements exclude lower-income users.	(244)
	Privacy & Data Security	Privacy fears reduce data sharing; especially critical in mental health domains.	(245, 246)
	Social Influence	Recommendations from peers or clinicians can increase trust and perceived legitimacy.	(247–249)
	Environmental Support	Training and support resources have been shown to reduce friction and improve sustained engagement.	(250)

Despite well-documented determinants of engagement, most DHIs still struggle to achieve and sustain meaningful user engagement, especially in real-world settings (56, 63, 65). Fragmented design efforts and limited implementation planning contribute to high attrition rates, with many users discontinuing use within days or weeks of initial download (22, 51).

### 2.3 Patterns of failure: common pitfalls in digital health engagement

In addition to the above-mentioned factors influencing engagement, DHIs are susceptible to recurrent “failure modes”, predictable or recurring patterns that prevent the digital solution from achieving its intended health, behavioral, or clinical outcomes potential (66). These include both early-stage engagement losses as well as deeper systemic issues. Addressing them is essential for transforming short-term curiosity into long-term value.

**Figure 1:**
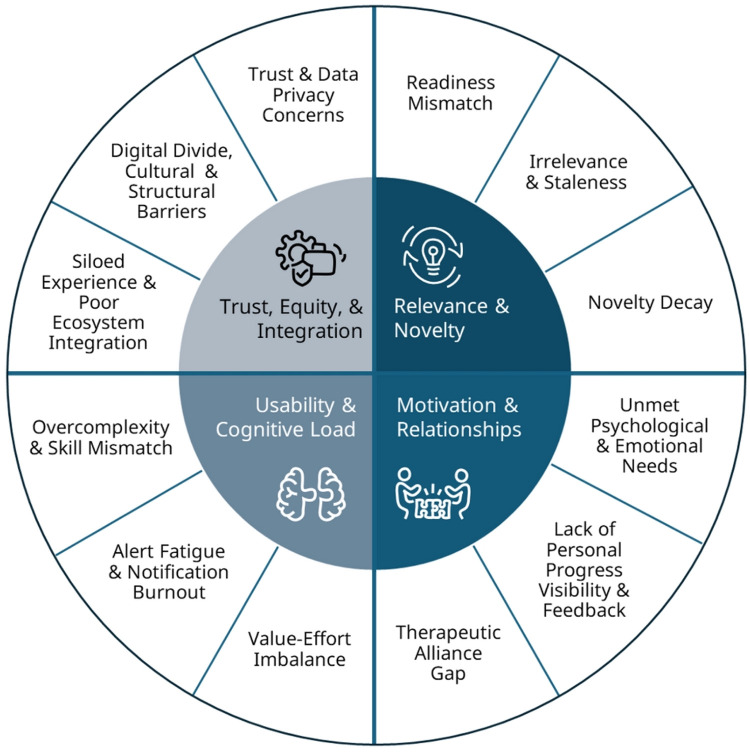
Common failure modes of DHIs.

Based on published data as well as practical experience, we identified several of these failure modes that can be categorized into four domains as shown in Figure 1.

#### 2.3.1 Relevance and novelty

Early engagement can be hindered by a *mismatch in readiness* as well as *irrelevance and staleness* of content and messages (35, 57, 67–69). DHIs often use a one-size-fits-all approach for prompts and content that overlooks users' varying and changing emotional and cognitive states. This can lead to frustration, fatigue, and disengagement, particularly when messages arrive during low-motivation or high-stress periods (67, 68). *Novelty decay*, the fading of initial interest in a new digital tool, has been well-documented as a cause of rapid disengagement and drop off in digital health solutions, often over 80% within the first week, when new value is not introduced (22, 51, 53, 70, 71). Just-In-Time Adaptive Interventions (JITAIs) can address this by tailoring support to contextual signals like mood, time, or location (35, 69). While underused due to design complexity, advances in affective computing and passive sensing are enabling more adaptive approaches (57, 72). Additional strategies include ecological momentary assessment (EMA), a tool for capturing time-varying behaviors, feelings and experiences in individual's daily routines (73), reinforcement learning for personalized timing, and behavioral activation during low-receptivity phases (74).

#### 2.3.2 Motivation and relationships

*Therapeutic alliance*, which has traditionally been conceptualized as the collaborative bond between client and healthcare provider, is another key predictor of adherence and clinical improvement in both traditional and digital interventions. Yet, many DHIs fail to implement the relational elements that underpin alliance, such as empathy, responsiveness, and attunement. Guided interventions (e.g., coaching, messaging, check-ins) consistently outperform standalone apps in both adherence and outcomes (75, 76), highlighting the need for such relational components. Relational design strategies, such as personalized messages, conversational agents, or avatars, can partially compensate for this experienced relational void, though they rarely match the perceived authenticity, empathy, or trustworthiness of human interaction (68, 77–79). For example, Fortuna et al. (80) found fully automated tools scoring lower on alliance metrics and faster drop-off. Hybrid models that offer light-touch human support, adaptive feedback, and preference-based personalization can better build and expand product-user alliance based on individual preferences and feedback (81). Motivational engagement decline can also be caused by *unmet expectations and needs*, often relating to the need for competency, autonomy, and relatedness (82). *Lack of early personalized feedback and visible progress* further erodes motivation beyond initial curiosity. Countermeasures include progressive goal setting, adaptive content, micro-reinforcements, habit-loop design, and gamification through rotating challenges and quests (35, 83, 84).

#### 2.3.3 Usability and cognitive load

Beyond these macro-level challenges, additional design-related failure modes further erode sustained engagement, including *value–effort imbalances*, o*vercomplex designs and user flows*, as well as *alert and notification fatigue* (66). Livieri and colleagues (85) identified key factors like time commitment, manual input processes, the need to remember passwords, logging in every time, and waiting for the loading process as burdensome enough to reduce users' interest. Mitigation strategies include progressive onboarding and graded tasks, adaptive notification logic, AI-curated content, empathy-driven language, interoperable system integration, and transparent privacy safeguards. These measures can preempt disengagement by making the intervention more doable, usable, and relevant for the individual user.

#### 2.3.4 Trust, equity and integration

On the ecosystem level, *structural barriers* like limited broadband access, restrictive data plans, and unstable or unsupportive living environments can significantly contribute to disengagement (86, 87). Furthermore, lower digital and health literacy, alco called the *digital divide,* remains another critical failure point to equitable DHI engagement, as shown by evidence that lower socioeconomic status (SES) groups show higher attrition rates, reduced engagement, and poorer outcomes (85, 86, 88). Many DHIs also lack *cultural tailoring and linguistic inclusivity*, further alienating underserved populations (89). In the telehealth context, these disparities are amplified by *trust and privacy concerns*, device limitations, and *siloed experiences* (90), which can further impede consistent participation. Besides privacy and security concerns, confidentiality, data ownership or lack of control over data, digital fraud, and the integrity of personal health data are often reported patient concerns (85). To address these, developers should consider participatory co-design, provide low-bandwidth solutions like SMS or offline alternatives, culturally inclusive content, and digital literacy support to improve contextual fit and reduce inequities. Non-digital augmentation, such as phone-based coaching, can further support adoption.

Addressing these failure modes requires shifting from reactive troubleshooting to anticipatory, user-centered design. A breakdown in the alignment of DHIs and user capability (e.g., literacy, skills), opportunity (e.g., access, social support), or motivation (e.g., perceived relevance), as described in the COM-B Framework (91), inevitably disrupts user engagement. Like other researchers (34, 56), we advocate for iterative co-design, continuous contextual adaptation, and ongoing evaluation to ensure interventions evolve alongside user needs. Embedding dynamic content, adaptive timing, relational cues, and equity-driven accessibility within the intervention architecture can transform short-lived novelty into sustained engagement, thereby maximizing both behavioral and clinical outcomes (92).

### 2.4 Measurement heterogeneity: challenges and imperatives in capturing digital health engagement

The accurate measurement of user engagement in DHIs is both critically important and methodologically underdeveloped (55, 93). Engagement has been variously defined as a behavioral, cognitive, emotional, or experiential construct, or combinations thereof (63). This theoretical ambiguity is reflected in the heterogeneity of measurement approaches. Ng et al. (94) found that fewer than 40% of DHI trials report both objective and subjective measures of engagement, and even within those that do, operational definitions vary widely. For example, some studies treat “retention” as the number of users who complete an intervention, while others define it by the duration of continued use or the number of completed modules (unpublished data, based on commercial product engagement definitions). This lack of harmonized metrics impedes efforts to synthesize evidence and develop predictive engagement models.

This inconsistent terminology (e.g., adherence, usage, uptake, stickiness) and methodological diversity (e.g., daily logins vs. feature-level interactions) can obscure the link between engagement and clinical efficacy and becomes particularly problematic when attempting to correlate usage patterns with behavior change or health improvements (18, 60, 95).

### 2.5 Metrics in practice: what we track vs. what matters

Digital health engagement is typically assessed through two fundamentally different categories: (a) objective behavioral metrics, which are automatically collected by most digital platforms and include usage volume and patterns, and (b) subjective self-report metrics, which capture the user's psychological experience, such as motivation, immersion, or affective involvement (45, 56). Neither alone provides a full picture (55).

Many studies still overlook dynamic, in-the-moment engagement fluctuations influenced by motivation, emotional salience, or context. More nuanced metrics such as engagement quality (i.e., depth vs. frequency), micro-level interaction patterns (e.g., pace of learning, latency between sessions), contextual engagement (e.g., time of day, concurrent stress, location, social setting), relational metrics (e.g., perceived alliance with an app or chatbot), or goal-concordant engagement (alignment with user-stated, patient priority goals) remain rarely implemented, despite their potential to illuminate how and why digital tools succeed or fail in real-world contexts.

Furthermore, passive sensing data (e.g., smartphone mobility patterns, screen interaction, speech cadence) and ecological momentary assessments (EMAs) can provide powerful insights into user context, affect, and readiness for engagement, but are still underused outside of academic settings (57, 96). A comprehensive list of digital health engagement metrics that either directly measure engagement components or predict outcomes can be found in Table 2.

**Table 2:** Behavioral and subjective measures of engagement.

Measures	Metric type	Description	Examples
Behavioral (objective) Measures	Log-ins/App Launches	Number of times a user opens the app or logs in	User logs into a meditation app three times per day
	Retention	Proportion of users who continue using the intervention over time	60% of users are still active after 30 days
	Dropout/Churn Rate	Percentage of users who stop using the intervention prematurely	25% of users stop using the app within the first week
	Frequency of Use	Number of sessions per day/week; regularity of use	User engages with the app five times per week
	Session Duration	Amount of time spent per session using the intervention	User spends 15 min per session on average
	Time between usages	Pace of use, time gaps between sessions, patterns of module completion	User completes modules in short bursts every few days
	Feature Usage	Tracking the use of specific features or modules within the app	User completes features like journaling, goal-setting, and mood tracking modules on a regular cadence
	Contextual Engagement	User engagement in relation to context (time, location, social setting)	Accelerometers, GPS, proximity sensors, or general usage in specific contexts (e.g., user interacts with the app mostly at night or during stress)
	Goal-Concordant Engagement	Alignment between app use and the user's stated personal health goals	Users log more steps after setting a step goal
	Physiological & Behavioral Adherence	Macro-engagement metrics that measure real-world health behaviors the app is targeting	Step/activity levels; medication adherence tracking; step tracker use & consistency; continuous monitoring (e.g., CGMs, HR monitors), screening cadence
	Engagement Patterns	Calculating engagement trajectory or patterns that could predict outcomes	Difference between “intended use” versus “actual use” (93); Latent growth model calculation (63)
	Dose-Response Model	Level or type of engagement required for clinical benefits	TWED components target, way, engagement, and dose to operationalize dose-response (55)
Subjective (user-reported) Measures	Loyalty & Satisfaction	Measures users' subjective value perception or satisfaction	Net Promoter Score (NPS); Customer Satisfaction (CSAT); Patient Satisfaction with Digital Tools (PSAT-H); mHealth App Satisfaction Questionnaire (MASQ)
	App Store Ratings	Rating and feedback provided through external platforms	Number and star rating of apple reviews; sentiment and praise of reviews
	In-the-moment surveys or prompts	User reports of mood, stress, context, motivation, or other perceptions	Ecological Momentary Assessment (EMA); custom questions or tracking prompts
	Usability	Measures of system usability	System Usability Scale (SUS), Consumer Effort Scale (CES), User Effort Score (UES)
	Engagement Depth	Quality of engagement as a combination of various components or flow experiences	User Engagement Scale (UES/ UES-SF); Digital Behavior Change Intervention Engagement Scale (DBCIES); Flow State Scale (FSS) adapted to digital contexts
	Self-Reported Engagement	User's subjective ratings of their involvement and engagement	Mobile-Centered Digital Health Readiness: Health Literacy and Equity Scale (mDiHERS); custom surveys of feeling involved and interested
	Emotional and Cognitive Engagement	Affective involvement, motivation and immersion based on validated measures	Positive and Negative Affect Scale (PANAS) adapted for digital interventions
	Motivation	Measuring interest, enjoyment, perceived competence, value, and relatedness	Intrinsic Motivation Inventory (IMI)
	Perceived Alliance	User's perceived therapeutic relationship with the tool or agent	Digital Working Alliance Inventory (DWAI) Scale; Session Rating Scale (SRS); mobile Agnew Relationship Measure (mARM)
	Trust	Trust in the effectiveness or safety of the digital solution, which could predict future engagement	Trust in Automated Systems Scale; E-Health Trust Scale; Digital Therapeutic Alliance instrument

### 2.6 Engagement vs. outcomes: a critical disconnect

A key challenge is linking engagement to meaningful clinical outcomes. High usage does not automatically translate to improvement, and some users may experience benefit with only minimal interaction, particularly in JITAI or habit-based models (56, 63, 97). Conversely, sustained usage without psychological involvement may reflect “click compliance” rather than therapeutic exposure (22).

This complexity underscores the need for dose-response models of engagement, in which researchers specify what level, type, or dimension of engagement is needed for clinical benefit, which features contribute most to change, and what engagement trajectories (e.g., bursts vs. consistent use) are associated with outcomes.

## 3 Conceptual foundations of the ENGAGE framework

**Figure 2:**
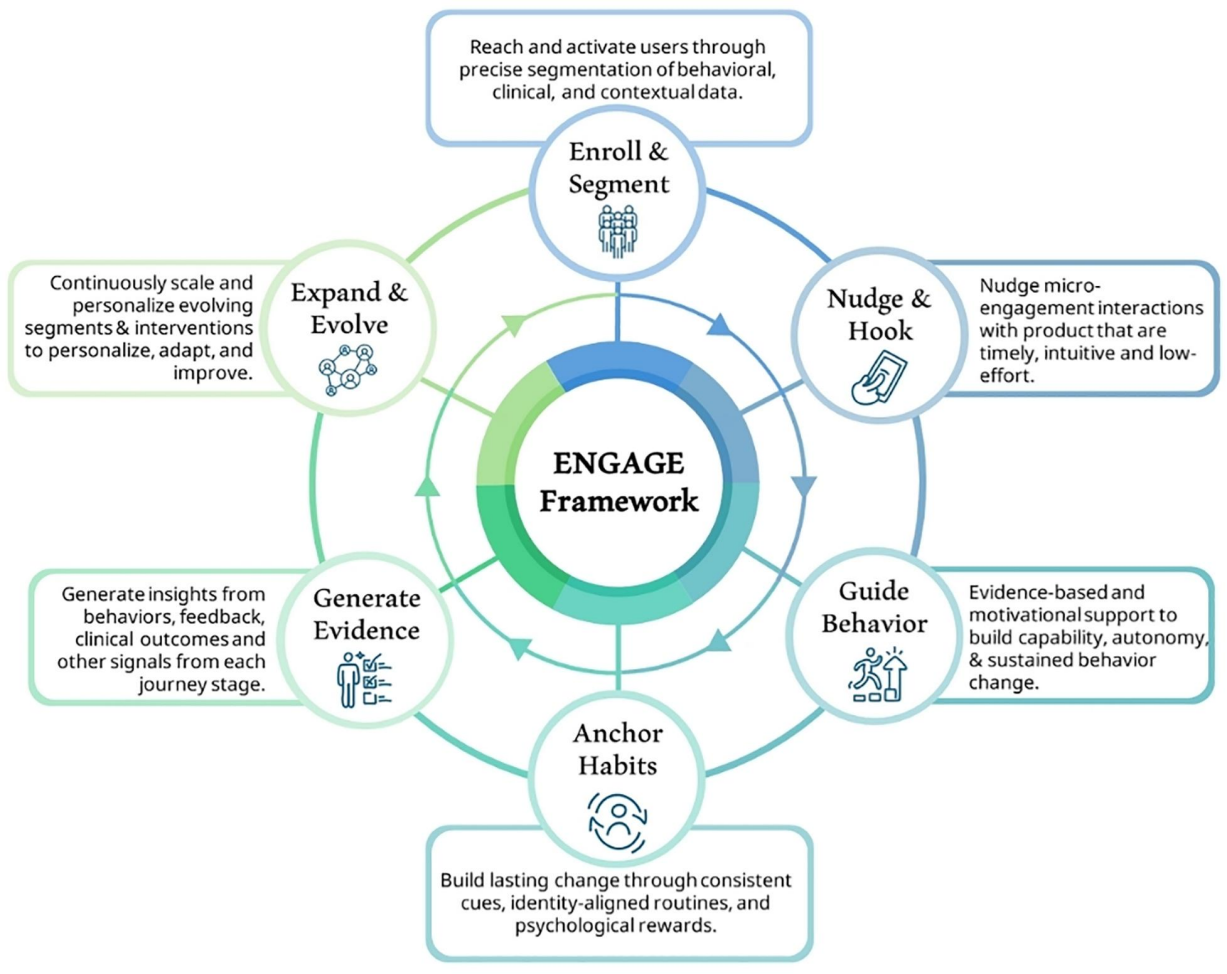
The ENGAGE framework.

The ENGAGE Framework synthesizes and integrates the above-mentioned critical advancements and frameworks, meeting the need for a unified, cohesive, and effective precision engagement framework. It also relies heavily on the COM-B Framework (Capability, Opportunity, Motivation—Behavior) and Behavior Change Techniques (BCTs) (43, 91), Self-Determination Theory (82), and Effective Engagement Framework (31), while also integrating other framework components with the goal of guiding DHI developers to create more impactful solutions to achieve meaningful clinical outcomes using a single, unified engagement framework. Figure 2 visualizes the cyclical architecture of the ENGAGE Framework from a high-level view, while Table 3 summarizes the individual steps' goals, applicable behavioral science theories and drivers, as well as example techniques and metrics.

**Table 3:** The six steps of the ENGAGE framework: theories and behavioral drivers, techniques, and example metrics.

Step & goal	Core behavioral science theories & drivers	Example techniques	Representative example metrics
1. Enroll & Segment	Salience Messenger Effect Ego Alignment Social Proof Loss Aversion Scarcity & Urgency COM-B Behavioral Targeting Fluency	Search Engine Marketing (SEM), Generative Engine Optimization (GEO), or Answer Engine Optimization (AEO) Patient-Similarity Networks (PSN) Personalized landing pages, lookalike modeling, peer storytelling & influencer campaigns, identity-based messaging Multilingual, multicultural Creatives Stratification & Segmentation (by readiness, health status, etc.) Intent-propensity models Onboarding flow optimization	Brand lift and awareness surveys Bounce rate Time on page Percentage of eligible population reached Engagement by segment Click-through rate (CTR) Upper funnel metrics like conversion-to-enroll or complete onboarding
Reach & activate the right users			
2. Nudge & Hook	Nudge theory Digital Therapeutic Alliance Reciprocity Defaults Ease of Use Self-Determination Theory (autonomy, competency & relatedness) JITAI Design Principles	User-centric experience design Just-In-Time-Adaptive-Interventions (JITAI) Chatbot/coach micro-interaction Tiny habits & micro-commitments Gamified feedback & incentives Personalized nudges Social proof banners Social support (human or AI-supported)	Time to first meaningful use Session return rate Feature activation rate Drop-off points & funnel conversion Satisfaction & Effort Scales Trust & alliance ratings
Convert curiosity and initial interest into consistent digital interactions (micro-engagement)			
3. Guide Real-World Behaviors	COM-B Self-Determination Theory (SDT) Behavior Change Techniques (BCTs) Goal-Setting Theory Social Cognitive Theory (SCT) Motivation Theories (MI, self-efficacy)	Goal setting & action planning Self-monitoring and tracking features Peer support/community forums Human or AI-guided coaching Choice architecture (e.g., defaults, framing) Motivational Interviewing Flows Gamified progression Graded tasks	Action plan completion Sensor-verified behaviors (e.g., steps, medication adherence) Care plan adherence Frequency of self-monitoring Community participation (% of users posting/responding)Real-world task follow-through Leaderboard placement
Drive real-world behavior change and macro-engagement			
4. Anchor Habits	Habit Loop Theory Variable Reinforcement Schedules Implementation Intentions Identity-Based Habits Behavioral Substitution Social Norms & Comparison	Streaks and badges Implementation intention prompts (“If-Then”) Contextual cues and reminders Social accountability (buddy system, leaderboards) Progress tracking Identity-affirming messages Just-In-Time-Adaptive-Interventions (JITAI) Gamification like badges, points, rewards, progress visualizations, or leaderboards	Usage consistency and longitudinal usage patterns Consecutive day/week streaks Habit strength scale (self-report) Repeated behavior in consistent context Reduction in need for nudges Substitution behaviors (e.g., water over soda)
Establish routines and embed health behaviors for long-term health impact			
5. Generate Evidence	Feedback Theory Framing Gradient Effect Self-Determination Theory (Competence) Cognitive Appraisal Theory	Personalized dashboards and visual insights (e.g., trend lines, comparisons) Progress feedback linked to user goals Mixed-method outcome collection (clinical, behavioral, PROs) Norm-based benchmarking	PRO scores (e.g., QoL, energy, stress) Clinical markers (e.g., PHQ-9, HbA1c) Insight view rate Goal progress tracking Perceived goal achievement Percentage of users completing feedback prompts
Measure outcomes, close feedback loops, and personalize value to users and systems			
6. Expand and Evolve with AI	Person-Action Fit Predictive Modeling Digital Phenotyping Equity-by-Design JITAI Frameworks COM-B Data Feature Mapping	Reinforcement learning (RL) Context-aware JITAI triggers Digital twins Sentiment detection from language use Next Best Action engines AI-guided timing/channel optimization Equity dashboards and bias monitoring	Precision/recall of prediction models Engagement uplift vs. static arm Response rate by timing/context Percentage of content/actions personalized Subgroup parity & fairness metrics Percentage of adaptive messages acted on
Adapt engagement based on what works for whom, when, and why for real-time optimization			

### 3.1 Step 1: Enroll & segment

Effective engagement begins before a user interacts with DHIs. This initial step focuses on strategically matching and enrolling individuals with the right solution, channels, and messages, as well as behavioral nudges using segmentation and personalization tools and behavioral insights. The aim is to build early trust, increase relevance, reduce stigma, and establish the foundation for digital therapeutic alliance.

Rather than one-size-fits-all campaigns and engagement journeys, successful outreach relies on data-driven segmentation to identify who will benefit most, when, and how. This process is dynamic, not a one-time action, and is the first step in a continuous cycle of precision personalization.

#### 3.1.1 Strategic segmentation and targeting

Matching users to interventions begins with precise segmentation and stratification based on (a) clinical data (e.g., diagnosis, disease profile, risk level, comorbidities), (b) demographic data [e.g., age, gender, geographic location, area deprivation index (ADI)], (c) sociocultural data (e.g., language, geography), (d) behavioral and psychological data (e.g., health goals, capabilities, behavioral drivers, specific pain points and barriers, priorities, expectations, motivation, readiness, concerns, mental models, beliefs, status quo behaviors or solutions), (e) eligibility information, and (f) engagement data (e.g., prior actions, website/email engagement, etc.). Incorporating sociocultural factors into outreach strategies has been shown to increase resonance, trust, and equity (1, 92, 98, 99). Thus, stratification allows for tailored outreach strategies based on what matters most to different groups and enables precision engagement by connecting individuals with the solutions best suited for them.

Enrollment must proactively address structural barriers such as digital redlining (the systematic exclusion or disadvantage of specific communities from digital access, services or technological benefits), language and literacy differences, and unequal device or broadband access. Segmenting by language, digital literacy, ADI, and sociocultural identity ensures outreach does not disproportionately exclude underserved groups. Representation should be monitored using eligibility-to-enrollment parity metrics.

A common computational segmentation method is building *patient-similarity networks (PSNs*), that organize patients into a network based on their multidimensional characteristics (100). This enables more accurate risk stratification, engagement strategies, and treatment recommendations, informed by outcomes in comparable patient groups (100–103). In the context of the ENGAGE Framework, PSNs can enhance initial segmentation by identifying “lookalike” users whose engagement patterns, health profiles, and intervention responses align, thereby informing targeted micro-engagement strategies from the outset. As the network grows, outcome data from similar patients (see Step 5: Generate Evidence) can further refine personalization over time (which is discussed in more detail as part of Step 6: Expand & Evolve), strengthening both predictive accuracy and real-world effectiveness.

#### 3.1.2 Predictive and data-driven outreach

Traditional outreach often relies on static, demographic-based lists, leading to irrelevant or poorly timed messages. Modern strategies use real-time signals and predictive models, such as value-centered marketing (VCM) and next best action (NBA) algorithms, to align content with user behavior, intent, and readiness. Dynamic outreach increases relevance by triggering engagement at high-salience moments (e.g., during portal login, appointment reminder, or lab result notification), significantly improving conversion and satisfaction. However, ethical data use is critical for earning user trust. Transparent consent processes, easy-to-understand privacy policies, and strong data safeguards (e.g., encryption, tokenization) ensure respectful engagement (104–108). To foster long-term relationships, DHIs must communicate how data is used and give users control over preferences.

#### 3.1.3 Contextual relevance and mindset matching

Capturing attention depends not only on the message but also on the timing and context. Individuals are more receptive to health messages during care-related activities, rather than at random moments. Aligning messages with the user's mental state (e.g., decision-making mindset) improves salience and cognitive fluency, making the message easier to process and act upon. Omni-channel strategies (i.e., delivering consistent, timely content across personal devices) can enhance perceived alignment and create cohesive user experiences. A strong digital presence, as well as search engine optimization and providing multilingual creative assets aligned with diverse populations, can increase the likelihood of meaningful engagement from the start.

#### 3.1.4 Personalization and identity-reinforcing messaging

Messages that reflect users' lived experiences and values, especially those that align with the individual's identity and their desired quality-of-life improvements, are more likely to resonate. Creating identity-reinforcing messages (e.g., “People like you are already taking steps to improve sleep health”) enhances ego alignment and leverages social proof, two powerful behavioral motivators (109, 110). The goal is to offer actionable value in the moment of need right from the start, rather than generic awareness campaigns.

#### 3.1.5 Behavioral science principles

Several other key behavioral science principles enhance this step. These include *Salience* (capturing attention through novelty, timing, and relevance), *Ego Alignment* (reinforcing self-image and making individuals feel uniquely seen), *Messenger Effect* (selecting trusted, relatable voices like fellow patients or providers), *Social Proof* (showing how others in similar situations are acting), *Loss Aversion* (emphasizing missed opportunities to prompt consideration) and *Scarcity/Urgency* (time-limited offerings or enrollment windows). These mechanisms can increase receptivity and motivation from the very first touchpoint. Relevant success measurements at this stage include click-through rate (CTR), conversion to enroll, brand lift and awareness surveys, bounce rate, time on page, percentage of eligible population reached and successfully converted, and engagement by segment.

#### 3.1.6 Other tools & tactics

Additional marketing and outreach tools include search engine marketing (SEM) and search-optimized landing pages, refined keywords from broad terms to exact-match keywords and phrases, peer storytelling and influencer campaigns, and multilingual, multicultural assets that reflect the diverse target audiences and segments.

#### 3.1.7 Common challenges

Despite its foundational role, implementing a precision-based outreach and enrollment strategy in DHIs is often hindered by fragmented data systems, limited technical capacity, and organizational silos. Many teams rely on superficial and static demographic segmentation (e.g., focused on names or demographics) or outdated mailing lists rather than being grounded in psychological relevance, sociocultural context, or behavioral and readiness-based targeting due to limited access to specific user data and real-time intent signals (63, 111, 112). Predictive modeling for personalized outreach (e.g., next-best action, dynamic segmentation) is often underutilized due to infrastructure gaps, including siloed data bricks and data interoperability challenges, or lack of data science resources. Additionally, teams often struggle to build omni-channel coordination across SMS, in-app/web messaging, portals, payment and insurance partners, and advertising platforms, limiting their ability to reach users in moments of receptivity (64). Privacy and consent complexities also limit access to sensitive but engagement-critical data like beliefs, readiness, or digital phenotypes (70). Finally, few teams invest in identity-based messaging or value-aligned framing, leading to outreach that feels generic and fails to build early trust or therapeutic alliance (56, 109). Being aware of these challenges can help to actively prevent or overcome them.

#### 3.1.8 Summary

The first step of the ENGAGE Framework lays the groundwork for long-term engagement by ensuring that the first touchpoints are targeted and the right people see the right message, at the right time, and in the right context. Precision outreach rooted in trust, respect, and behavioral science helps to begin to turn awareness into action**.**

### 3.2 Step 2: Nudge & hook—driving micro-engagement

Once awareness and relevance are established, DHIs must convert initial curiosity into actual digital interactions. This step, often referred to as the “hook,” focuses on initiating micro-engagement: short, strategic interactions that build early momentum, trust, and emotional investment. Micro-engagement goes beyond getting users to click or enroll and extend to building familiarity and usage patterns, creating positive expectations, and reinforcing that the tool is relevant, useful, and easy to integrate into daily life.

#### 3.2.1 Designing for readiness

Crucially, this stage must account for readiness, a dynamic state shaped by motivation, stress, cognitive bandwidth, and emotional safety (113, 114). Systems that ignore readiness signals risk user disengagement or psychological reactance (67). Instead, adaptive designs can detect and respond to behaviors such as hesitancy or indecision (e.g., lingering on FAQ pages or selecting “maybe later” prompt answers) by offering low-pressure pathways or contextual reassurance, rather than repetitive calls to action (65, 71). Available assessments to measure digital health readiness include the Mobile-Centered Digital Health Readiness Scale (mDiHERS) (115), the Digital Health Readiness Screener (116), the Digital Health Readiness Questionnaire (DHRQ) (117), the eHealth Literacy Scale (eHEALS) (118), and the Conversational Health Literacy Assessment Tool (CHAT) (119). All these assessments aim to better assess digital health readiness, including literacy and equity, to ensure users are capable and ready to effectively use the offered digital health services. Since readiness is fluctuating and non-static, personalization based on readiness must be real-time and respectful. Pushing static goals despite signs of user fatigue or ambivalence undermines trust and engagement. Readiness-sensitive design throughout the user experience and product flow bridges the gap between user behavior and intervention, enabling personalization that aligns with the user's evolving state.

#### 3.2.2 Building digital therapeutic alliance

A key psychological driver at this stage for building long-term engagement is Digital Therapeutic Alliance (DTA), a term used to describe the user's perceived connection to the digital tool. Research shows that DTA, which is built through empathy, responsiveness, and shared goals, is emerging as a promising concept that can enhance the effectiveness of digital interventions by fostering user engagement and adherence through goal alignment, task agreement, usability, non-judgmental responses, bond development and perceived empathy (120–125). Although quantifying DTA remains a challenge (126), it can be supported through features like personalized onboarding, coach interactions, and conversational interfaces, and measured through trust and alliance scales, like the Digital Working Alliance Inventory (DWAI) and Mobile Agnew Relationship Measure (mARM), as well as user satisfaction ratings (e.g., NPS, CSAT) (127, 128).

Early engagement varies widely across literacy, language, and cultural groups. Thus, outreach messages should be multilingual, low-literacy, and culturally aligned to avoid inequitable early dropout. Micro-engagement should be tracked through parity metrics (e.g., open rates and return sessions) across demographic and SDoH subgroups.

#### 3.2.3 Emotional salience and identity alignment

Multiple studies show emotional and identity-based cues foster loyalty and advocacy. For example, pleasant and arousing experiences on engagement platforms can lead to increased brand loyalty (129), while integrating emotion recognition in usability testing can enhance web application UI design by providing a more complete and objective picture of user experience (130). Additionally, it has been shown that social identity salience affects group-based emotions, highlighting the importance of group-based appraisals (131). When interventions affirm participants' identities, such as through self-affirmation exercises or demographically matched role models, engagement and positive outcomes are heightened, especially in contexts where identity threats are present (132).

#### 3.2.4 Behavioral science principles

To encourage micro-engagement, small, frequent interactions within the DHI, like quick check-ins, tracking, and notification-driven interactions, help to bring users back into the digital platform and guide them toward valuable features. Ease of use, reciprocity, defaults, social proof, positive emotion, and micro-commitments help to pull the user into the digital experience, while quick wins like badges, hope-framed success stories, or peer comparisons (e.g., “8,375 people logged in today”) create positive emotions and reinforce both competence and motivation. Objective metrics examples include time to first meaningful use, return session rates, feature interaction frequency, and drop-off analysis, while subjective metrics examples include System Usability Scale (SUS) and consumer effort scale (CES).

#### 3.2.5 Other tools & tactics

Successful strategies combine behavioral cues, JITAIs, and emotionally resonant design to reduce friction and encourage repeat use. These interactions (e.g., mood logs, goal pledges, or wearable-triggered prompts) serve as behavioral scaffolds that build familiarity and support progression toward sustained use (64, 133). Personalization, intuitive user interface (UI) and user experience (UX), and thoughtful defaults help reduce cognitive load and remove early barriers to engagement (134).

#### 3.2.6 Common challenges

Designing effective micro-engagement requires precise timing, contextual adaptation, and emotionally intelligent user experience design, all of which pose challenges for many product teams. A major barrier is the absence of real-time behavioral or contextual data to trigger just-in-time nudges, and most DHIs cannot detect when a user is emotionally or cognitively ready to engage. Personalization engines are often static, using generic intake data instead of evolving signals like click patterns, delay behaviors, or passive indicators of readiness. Resource-constrained teams may lack the infrastructure for adaptive messaging, reinforcement learning, or even robust A/B testing (57). Emotional salience, identity alignment, and DTA are frequently overlooked in favor of fast, but transactional UI/UX, leading to micro-engagement that is technically functional but psychologically flat. Many products also overuse reminders without sensitivity to timing or tone, triggering user reactance or fatigue (22, 67). Finally, the measurement of micro-engagement tends to focus on clicks and session duration, with little insight into affective involvement, motivation, or friction points, leaving critical feedback loops unclosed (63).

#### 3.2.7 Summary

Step 2 involves emotionally intelligent activation using personalization, behavioral science, and real-time data to build early trust, engagement, and satisfaction. Micro-engagement involves the strategic use of behavioral cues, interactive nudges, and JITAIs to sustain attention, prompt action, and create a satisfying, frictionless experience. This step builds the psychological and behavioral foundation for deeper engagement, anchoring the user's experience in trust, relevance, and reward. It transforms momentary curiosity into sustained connection, which is essential for the downstream success of any DHI.

### 3.3 Step 3: Guide real-world behaviors

Many DHIs succeed at capturing in-app attention but fail to drive sustained behavior change in users' daily lives outside the digital platform. Step 3 focuses on bridging that gap by translating digital interactions into consistent, real-world health skills, decisions, and behaviors that improve clinical outcomes.

#### 3.3.1 Translating clicks into real-world behavior

Where Step 2 (i.e., micro-engagement) emphasizes digital interactions, Step 3 (i.e., macro-engagement) ensures that users apply the acquired knowledge and skills outside the platform. As described earlier, engagement that doesn't extend into real-life behavior is insufficient to produce clinical benefit (31). Real-world behavior is the endpoint that digital health should ultimately impact. For example, reading about nutrition is not the same as eating a healthier diet. Similarly, setting up medication reminders is not equivalent to taking the medication consistently. Without this translation, engagement is unlikely to yield meaningful outcomes (31, 135). Macro-engagement is the essential mechanism through which intention becomes action, and digital health becomes health behavior.

#### 3.3.2 Designing for macro-engagement

Achieving this shift requires applying evidence-based behavior change techniques (BCTs), such as goal setting, self-monitoring, feedback, and action planning, within robust theoretical frameworks like COM-B, Self-Determination Theory (SDT), and PRIME Theory (25, 82, 91, 135). These models help identify and address key behavioral barriers within domains like lack of capability, limited opportunity, or unstable motivation. To design for macro-engagement, these insights must be applied not just to what happens *in the app*, but what happens *because of it*. Macro-engagement is best assessed through a combination of factors, including feature activation metrics (e.g., goal progression, action plan completion, care plan adherence, self-monitoring frequency), which give insights into how solution features are used to support real-world implementation (Table 4).

**Table 4:** Macro-engagement strategies and techniques and their potentially associated mechanisms of action.

Strategy/technique	Description	Mechanism of action
Goal Setting & Action Planning	Supporting users in setting clear, personalized health goals and planning steps; helping to anticipate barriers and find solutions, as well as translating goals into concrete when/where/how plans.	Enhances clarity, increases perceived attainability, strengthens intention formation, promotes implementation planning, and increases perceived attainability.
Self-Monitoring & Feedback	Enabling users to track key behaviors (e.g., steps, sleep, medication) and receive feedback and progress updates.	Reinforces self-efficacy, strengthens behavior-outcome links, creates positive reinforcement and motivation through competence-building.
Motivational Interviewing (MI)	Using guided digital and human dialogue to explore ambivalence and strengthen value-based motivation.	Enhances reflective motivation and autonomy while reducing resistance. Aligns goals with intrinsic drivers.
Peer and Community Support	Creating a sense of belonging and shared experience through patient forums, peer stories and groups, or informal support.	Leverages social proof, boosts relatedness and accountability, reinforces social norms, and increases accountability.
Coaching and Human Touchpoints	Providing real-time or asynchronous coaching (human or AI-supported) to guide behavior change.	Builds therapeutic alliance, increases trust, enhances trust, and delivers personalized and problem-solving support.
Framing and Emotional Priming	Presenting content using emotionally resonant visuals, narratives, or success stories to drive action, engagement, and behavior change.	Activates emotional processing, increases memorability, and reduces cognitive friction.
Personalization & Feedback	Adapting content and timing to user preferences, behaviors, and context over time.	Improves perceived relevance, deepens engagement, supports sustained motivation and behavior change.
Use of Behavioral Frameworks	Applying models like COM-B, SDT, or PRIME to guide intervention design and personalization.	Enables barrier diagnosis and tailored strategy selection based on behavioral science.
Digital Therapeutic Alliance (DTA)	Fostering trust, empathy, and goal alignment via design or coach-user interactions.	Simulates relational support found in therapy, improving engagement and adherence, replicates components of in-person alliance.
Social Modeling and Accountability	Highlighting peer behaviors and use role models to encourage action and shared goals; leveraging social proof to drive commitment and norm-based behavior.	Reinforces social norms, reduces isolation, and boosts behavior through accountability.
Gamification	Leveraging game elements like points, leaderboards, and rewards to increase user engagement and motivation.	Creates interactive engagement, elicits positive emotions, and increases enjoyment. Clear contingencies and progress signals (milestones), as well as rotating challenges and mastery goals elevate intrinsic motivation.

#### 3.3.3 Context makes or breaks DHIs

Real-world behavior changes demand more than theory or app design alone. It requires integration into users' daily social, psychological, and environmental contexts. Supportive physical and social environments have consistently been shown to promote healthier behaviors and better health outcomes, while disadvantaged or unsupportive environments can hinder positive health choices (88, 136, 137). For example, Liu and colleagues (136) reported that better perceived neighborhood environments significantly promote physical activity and reduce sedentary behavior, smoking, and alcohol consumption, affecting life satisfaction both directly and indirectly. Meanwhile, living in a disadvantaged neighborhood is associated with increased mortality, especially in individuals of low SES (88). A favorable social environment, including individual-level social environment and residing in a neighborhood with greater community well-being, has also been significantly associated with ideal cardiovascular health (137). Thus, strategies must extend beyond the screen to include social support systems, real-time feedback loops, emotionally resonant framing, and environments that make healthy choices easy. Measuring community participation (e.g., forum activity, coaching responses) provides evidence of deep and active engagement that indicates a deeper level of implementation (138). Since real-world behavior change is shaped by structural inequities, including neighborhood safety, transportation, caregiving demands, and cost constraints, adaptive pathways and goal-setting should account for SDoH differences, with behavior-adherence equity monitored across groups.

#### 3.3.4 The role of social scaffolding and motivational support

When digital tools are supported by community, coaching, and well-designed defaults, users are more likely to sustain health behaviors over time. Research strongly supports that digital tools are most effective for sustaining health behaviors when combined with coaching, community support, and user-centered defaults. These elements drive engagement, accountability, and long-term behavior change across diverse populations and health conditions (139–141). Social scaffolding, including peer groups, caregiver involvement, or patient communities, enhances relevance, accountability, and motivation. Studies show that peer support increases long-term adherence to behavior routines (138, 142). Likewise, light-touch coaching and access to care teams or digital coaches (human or AI-driven), provide guidance and reinforcement, and enhance personalization and trust through DTA (121, 143). Similarly, features such as emotionally framed content, pre-selected defaults, and affective visuals reduce cognitive burden and prime users for action (134, 144). When paired with motivational interviewing techniques, such framing can activate motivation, especially for ambivalent users (145).

#### 3.3.5 Personalization for emotional resonance

Similar to Step 2, emotional resonance, identity affirmation, and contextual relevance at this stage can enhance sustained engagement and intervention impact. Interventions that evoke positive emotions (e.g., interest, inspiration) or use emotionally engaging content (such as storytelling or metaphor) have been shown to increase cognitive and affective engagement, making participants more likely to stay involved and benefit from the intervention (65, 146). Emotional engagement can be further deepened through personalization, ensuring that users' goals, communications, and experiences are tailored to their context, values, and readiness state (57, 63).

#### 3.3.6 Gamification

Gamification, using game elements like points, leaderboards, and rewards, has been shown to increase user engagement and motivation in digital health interventions across various domains, including chronic disease management, physical activity, and mental health (147). These elements foster motivation, adherence, and knowledge retention, and can make health-related behaviors more enjoyable and interactive (148–150). According to Fleming et al. (60), translating traditional evidence-based interventions into computer gaming formats has considerable potential for increasing the impact of online interventions. It has also been shown that online health communities and mobile apps that used gamification strategies resulted in higher user engagement and positive user experiences (151, 152). However, there are few independent trials and direct comparisons of game-based and non-game-based interventions, and the long-term sustainability of these effects is less clear, with many studies reporting that engagement and behavior change may decline as the novelty of gamification wears off (150, 153). In summary, gamification in digital health improves health outcomes by boosting engagement and driving short-term behavior change. However, evidence for gamification remains mixed, with effects varying by context and user group and with emerging ethical concerns around reward schedules and compulsive-use dynamics (154), and long-term effectiveness requires optimal design strategies and techniques for creating and embedding routines and habits into users' daily lives. Ultimately, real-world implementation should be supported by adaptive technologies that tailor interventions based on user goals, readiness, and evolving needs.

#### 3.3.7 Other behavioral science principles

When interventions are designed to not only inform but to activate, reinforce, and sustain behavior outside the digital experience, they are able to translate digital behaviors into real-world behaviors in the specific individual's context. Crucially, macro-engagement is strengthened through several behavioral science techniques. For example, feedback loops that tie user actions to personalized outcomes are strong drivers. When users track behaviors (e.g., steps, sleep, medication adherence) and receive timely, goal-linked feedback, it reinforces perceived competence, motivation, a sense of progress, and self-efficacy, which are key drivers of long-term change (63, 133). Behavioral execution [e.g., wearables data, medication possession ratios (MPR), diet logs, sleep and exercise data, behavioral tracking data] are behavior measures that can establish a causal relationship between micro- and macro-engagement, while adherence & retention metrics (e.g., follow-through on behavior prompts, 30-day retention, sustained care plan usage) indicate the sustained nature of user engagement.

#### 3.3.8 Common challenges

Despite strong theoretical support, and similar to the other steps, macro-engagement strategies are often under-implemented in DHIs due to structural, technical, and organizational barriers. Resource constraints and lack of embedded behavioral science expertise leads to limited application of frameworks beyond surface-level features. This often results in retrofitted and isolated techniques that are not mapped to users' specific barriers or motivations. Technical limitations (e.g., static content architectures, absence of adaptive logic) make it difficult to personalize experiences in real time or respond to evolving user needs. Data fragmentation further impedes the ability to connect digital behaviors with real-world outcomes, as few systems integrate across Electronic Health Records (EHRs), wearables, and self-reported tracking (57). Additionally, macro-engagement strategies are difficult to evaluate, requiring validated behavioral metrics that go beyond logins or clicks, and many teams lack the infrastructure or incentive to capture them. Even when data is available, privacy and regulatory concerns often restrict the use of contextual or biometric signals that could support timely behavioral nudges. Finally, commercial priorities often favor short-term engagement metrics [e.g., daily active users (DAU), retention] over long-term outcomes, creating misalignment between product goals and evidence-based engagement design. Together, these barriers limit the translation of digital interactions into sustained, meaningful behavior change.

#### 3.3.9 Summary

Step 3 turns behavioral knowledge into behavioral changes. It moves beyond just designing persuasive digital experiences toward enabling real-world execution. An alignment between internal drivers (motivation, capability, identity) and external scaffolds (social equity by dessupport, environment, system design) is needed to allow DHIs to progress from engagement metrics to meaningful impact. Strategies and techniques to achieve that include (a) applying evidence-based behavior change models, (b) building adaptive, feedback-driven interventions, (c) supporting users with social scaffolding and meaningful touchpoints, and (d) creating environments where healthy behaviors are easy and desirable. Ultimately, Step 3 translates intention and digital interactions into behaviors outside the app, which is necessary to produce lasting clinical value.

### 3.4 Step 4: Habit building

Establishing behaviors like taking medication, eating more fiber, or walking daily is only effective if they are repeated consistently enough to impact health outcomes. One-off actions, like eating a salad once a month, are insufficient to improve weight, HbA1c, or blood pressure. Therefore, DHIs must support the transition from conscious, effortful, initial behavior execution to automatic, sustained routines. This is the core goal of Step 4.

#### 3.4.1 From engagement to automation

Unlike short-term engagement strategies that rely on novelty or external rewards, habit formation requires stable cues, low cognitive effort, and repeated behavior in varying contexts (83, 155). On average, forming new habits takes 2–3 months, depending on the complexity of the behavior and individual factors (83). This highlights the need for long-term support strategies and tools to scaffold this trajectory by creating predictable, low-friction experiences that embed behavior within users' real-world environments.

#### 3.4.2 Reducing real-world friction

Designing for habit resilience also means addressing real-world barriers: time constraints, forgetfulness, and competing demands. Interventions that use implementation intentions (a pre-determined, self-regulatory plan that links a specific situation to a specific goal-directed response, thereby establishing an “if-then” plan), adaptive nudging (e.g., location-based hydration reminders), or low-barrier success strategies (e.g., streak tracking, graduated goal-setting) can help users sustain behaviors in imperfect environments (156–158). Additionally, DHIs should offer low-effort, flexible cues (SMS, voice reminders, culturally relevant anchors) and monitor habit stability across demographic and literacy subgroups.

#### 3.4.3 Behavioral science principles

Multiple behavioral principles can help design effective habit building features and real-world generalization. Cues anchored to time, location, or prior behavior (e.g., bedtime reminders, post-meal nudges, morning check-ins) help trigger actions and reinforce the cue-behavior-reward loop (64, 159, 160). Positive reinforcement, particularly variable schedules (e.g., intermittent feedback or “surprise” rewards), can strengthen the memory trace between action and reward, and improve long-term adherence (161). See Table 5 for more details. To evaluate habit formation, metrics could include behavioral consistency (e.g., repeated actions in stable contexts), streaks and goal progression (e.g., multi-day adherence patterns), unprompted behaviors and feature usage (e.g., frequency of logging, device interaction beyond initial novelty), and behavior substitution patterns (e.g., replacing sugary drinks with water). These indicators offer insight into whether behaviors are becoming automatic and, thus, sustainable.

**Table 5:** Techniques, frameworks, and example references for habit formation through digital tools.

Technique	Description	Mechanism of action	References
Adaptive Nudging	Context-aware prompts that adapt content, timing, and channel to receptivity and state (e.g., JITAI logic; RL-informed “next best action”). Trigger behavior using real-time data like GPS, time of day, or prior use.	Person-action fit increases response probability Just-in-time delivery targets moments of receptivity, need, and opportunity Learning systems personalize over time from feedback systems	(35, 69, 186)
Autonomy-Supportive Design	Embed autonomy principles through meaningful choices, non-controlling language, opt-ins, rationales, value alignment, privacy controls, customization, and optional features to foster self-determined motivation.	Supports psychological needs of autonomy, leading to increased intrinsic motivation and internalization Perceived choice and rationale reduce reactance and dropout, while increasing persistence and quality engagement Enhances perceived ownership of goals and paths, which sustains long-term engagement	(82, 157, 251–253)
Contextual Cueing	Deliberately embed stable, salient cues in the user's natural context (time, place, preceding action, object, social setting) to trigger the target behavior with minimal deliberation.	Stimulus-response learning and automaticity through repeated pairing of context and action to create habit loops Context stability and salience speeds retrieval and reduces reliance on motivation Prospective memory offloading and fewer intention-behavior gaps	(103, 166, 170, 171, 177)
Cues	Deliberately place stable, salient prompts in the user's natural environment (time, place, preceding action, object, social setting) to trigger the desired health behavior with minimal conscious effort.	Stable context–response pairings build habits by shifting control from intention to environment Consistent cues enhance automatic retrieval, reducing reliance on motivation, while “If–then” plans link cues to actions, boosting execution speed and likelihood External prompts offload memory and close intention–behavior gaps	(166, 167, 171, 177)
Gamification & Progress	Use points, levels, challenges, and narrative arcs to make progress visible and practice engaging. Align mechanics with meaningful health goals.	Clear contingencies and feedback loops support competence Variable challenges sustain interest, while mastery goals foster intrinsic motivation Progress visibility reinforces persistence Dopamine-mediated reward boosts motivation	(69, 161, 162, 183, 184)
Identity-Based Messaging	Use affirming language that links behavior to self-image by framing content around users' roles and identities (e.g., “athlete-parent,” “heart-healthy”), with nouns, role labels, and matched examples.	Identity salience translates behavior-based messaging into identity goals (“who I am/ want to be”) Ego-alignment and social proof increase relevance and commitment Reduces stigma and threat and increases message receptivity Promotes internalization of the behavior	(127, 128, 185, 186)
Implementation Intentions	Frame behavior plans using “if-then” scenarios to improve follow-through.	Automates initiation at cue presentation Shields goals from distraction Speeds up action selection and reduces executive burden Increases probability and timeliness of behavioral execution in complex environments	(156, 258, 259)
Low-Effort Behavior Design	Simplify the desired action to reduce friction and enhance repeatability using defaults, one-tap flows, pre-filled choices, reduced steps, and environmental restructuring.	Friction reduction lowers activation energy and increases follow-through Defaults exploit status-quo bias and choice architecture Cognitive load reduction improves execution under busyness and stress	(61, 150, 169, 176, 189, 190)
Progress Tracking & Streaks	Enable easy self-monitoring and display progress and optional streaks or badges to recognize consistency and build momentum.	Builds competence, self-awareness, and feedback sensitivity Control theory loop reduces uncertainty through monitoring, comparing, and adjusting Competency signals and achievement strengthen self-efficacy and habits Goal-gradient effect enhances motivation by boosting perceived progress	(254, 262)
Rewards	Provide timely reinforcement (intrinsic, social, or extrinsic) contingent on the target behavior, like in-app feedback, streaks/badges, small incentives.	Rewards boost behavior frequency and persistence Variable and uncertain payoffs heighten learning and sustain engagement Immediate, goal-linked rewards build competence and momentum Mastery- and values-framed rewards protect intrinsic motivation	(161, 263, 264)
Social Norms & Accountability	Leverage social norms (what others do or approve) through making advantageous peer behavior visible, and create accountability through check-ins, buddy systems, leaderboards, or public pledges.	Social proof, peer modeling, social comparison, and normative influence guide choices under uncertainty and increase motivation Observational learning and modeling boost self-efficacy Supportive accountability increases adherence	(133, 162, 174, 265, 266)
Variable Reinforcement	Provide timely, meaningful reinforcement (intrinsic, social, extrinsic) contingent on behavior; mix fixed with variable schedules (e.g., surprise badges, randomized “wins”) to sustain responding.	Operant conditioning shapes behavior Reward-Prediction error strengthens memory trace between behavior and reward Immediate, goal-linked feedback strengthens competence and momentum	(161)

#### 3.4.4 Other tools and tactics

To foster autonomy, competence, and relatedness, which predict sustained internal motivation according to Self-Determination Theory (82), simple tactics like user-selected goals, visual progress tracking, or community leaderboards can increase engagement while satisfying these psychological drivers. Additionally, complex behavior change is more achievable when broken into small, incremental steps. Techniques like graduated goal setting, streak tracking, and early wins increase perceived self-efficacy and motivation (158, 162). DHIs can scaffold success by designing low-barrier entry points and showing visible progress. Additionally, habit formation should not rely on coercion or willpower, but rather on evidence-based design principles that align with users' psychological needs, contextual realities, and capacity for behavioral change. Effective DHIs go beyond short-term nudges by embedding target behaviors into users' routines, environments, and identities, thereby facilitating automaticity and long-term sustainability. By anchoring behaviors within meaningful personal contexts, the most impactful interventions foster lasting health outcomes that persist beyond the duration of the program.

#### 3.4.5 Common challenges

Building habits through digital health interventions requires more than repetition: it requires careful orchestration of cues, timing, rewards, environment, and identity alignment (13, 163, 164). However, few products are designed with the behavioral precision or infrastructure needed to support this. Developers often lack the tools to implement habit loops, failing to create stable, repeated action patterns through consistent cues, routines, and rewards that reinforce behavioral automaticity (83, 165, 166). More importantly, most DHIs are built for episodic engagement, rather than designing for frequency, consistency, and friction reduction over time (90). Products also struggle to align incentives with intrinsic motivation. For example, external rewards may boost short-term use but crowd out the autonomous motivation needed for durable change (82, 167). If commonly used incentives like sign-up coupons, gift cards, or raffle entries are not used carefully, they might increase immediate engagement responses, but quickly loose users afterwards. Lastly, most developers do not evaluate or track behavioral consistency over time, relying instead on aggregate usage, which obscures habit strength or automaticity development (149).

#### 3.4.6 Summary

Ultimately, Step 4 is not about increasing users' willpower but about smart, respectful design that honors users' psychology, context, and capacity. By anchoring new behaviors to meaningful cues in real-world contexts, and reinforcing them over time, DHIs can convert initial engagement into behavior change, and ultimately, lasting health outcomes.

### 3.5 Step 5: Generate evidence

To drive meaningful impact, DHIs must not only engage users but demonstrate sustained improvements in health, behavior, and experience. Step 5 centers on measuring outcomes that matter (i.e., to users, providers, payers, solution clients, and systems) across diverse populations and real-world contexts, understanding what works, for whom, in what context, and ideally why. It is structured to support empirical validation by integrating UX interaction metrics, EMA and wearable-derived physiological signals, and clinical markers into a unified outcomes architecture that links micro-engagement to real-world and clinical impact. This process is essential not only for proving efficacy and causal real-world outcomes, but also for informing personalization, product iteration, and adaptive intervention design.

#### 3.5.1 From engagement to meaningful outcomes

Linking user engagement to health improvements serves both internal (e.g., product and clinical) and external (e.g., user-facing insight and motivation) functions. Measurement, which happens at each individual step, is linked at this stage to meaningful outcomes to prove impact, inform improvement, and drive personalization iterations for new and existing users. This requires capturing a broad spectrum of metrics: from symptom improvement and real-world health behaviors to psychological well-being, functional status, and user satisfaction (39, 168). Moreover, outcomes data should inform adaptive systems, so that interventions evolve based on what works for whom under what circumstances (64). Without measurement, personalization cannot mature and evolve beyond initial assumptions.

#### 3.5.2 Key metrics

Measurement must move beyond traditional endpoints and embrace a multidimensional approach. Effective strategies assess clinical change, behavioral execution, patient experience, and intervention-context fit. Table 6 lists these measurement dimensions in more detail. Clinical measurements include biomarker improvement (e.g., HbA1c, blood pressure, weight change), symptom reduction (e.g., PHQ-9 or GAD-7 scores), and treatment and medication adherence. These indicators are core metrics to demonstrate efficacy and are increasingly integrated into measurement-based care (MBC) frameworks (39, 169).

**Table 6:** Measurements to capture clinical, behavioral, and subjective outcomes, and contextual factors. .

Metric dimension	Description	Example metrics
Clinical Outcomes	Track symptom improvement, biomarker changes, and medication adherence using validated tools and longitudinal measurement.	**Metabolic:** HbA1c (%), fasting plasma glucose, CGM time-in-range, weight % change, BMI, waist circumference **Cardiovascular:** systolic/diastolic blood pressure (mmHg), LDL-C/HDL-C/TG, resting HR, ASCVD risk score change **Mental health:** PHQ-9, GAD-7, ISI (insomnia), PSS-10 (stress), remission/response rates **Safety and Utilization**: medication adherence (MPR, PDC, refill on-time %), adverse events, ED visits, hospitalizations, appointment adherence
Behavioral Outcomes	Monitor sustained behavior changes like physical activity, diet, or medication use via digital or sensor-based tracking.	**Physical activity:** steps/day, MVPA minutes/week, sedentary minutes, flights of stairs **Sleep:** duration, sleep efficiency, sleep onset latency, regularity index **Medication & routines:** % on-time dose, sensor-verified behaviors (smart caps), morning/evening routine completion **Diet & substance use:** servings/day (fruit/veg), added sugar grams, alcohol (number of days above limit), smoking (cigarettes/day)
Patient-Reported Outcomes (PROs)	Capture subjective experiences such as sleep, mood, energy, and quality of life, aligned with user goals.	**PROMIS domains**: global health, physical function, fatigue, pain interference, sleep disturbances, Quality of Life Scale (QOLS) **Targeted PROs**: perceived health status, energy/vitality, satisfaction with progress
Engagement & Experience Metrics	Assess usability and perception of the intervention through retention, dashboard interaction, NPS, or feedback engagement.	**Usage and flow:** Daily/ Weekly/ Monthly Active Users (DAU/ WAU/ MAU), session count, session return rate, time to first meaningful use, feature activation rate, funnel conversion & drop-offs **Perception:** NPS, CSAT, SUS, CES, DWAI/mARM (alliance/trust) **Content interaction:** dashboard insight view rate, dwell time on insights, click-through rate on recommendations, notification open/action rates, module completion, streaks, review ratings
Personalization & Contextual Fit	Tailor measurement to reflect differences in readiness, literacy, digital preference, and contextual factors.	**Personalization intensity**: % of content/actions personalized, response rate by timing/context, recommendation click-through, next-best-action uptake, % adaptive messages acted on **Equity & access**: subgroup parity/fairness metrics, equity dashboard flags, language coverage, device/bandwidth compatibility, literacy/readability match **Fit signals:** preferred channel adherence, opt-out/deferral patterns, help-request rates during key flows
Feedback Framing & Gradient Effects	Present outcomes as part of a continuum to reduce discouragement and support sustained effort.	**Feedback**: % of metrics shown as trends vs. binaries, average weekly slope from baseline, moving-average change, small-wins count (micro-improvements logged), time-in-improvement band, insight view rate **Mindset/Engagement**: prompts delivered in relation to acknowledged, “setback-recovery” streaks, reframing usage when plateaus occur
Goal Congruence & Personal Relevance	Link feedback to the user's personally defined goals for greater meaning and motivation.	**Goal-Congruency**: Goal-attainment scaling (GAS) t-scores, goal progress tracking, perceived goal achievement rating, % feedback items explicitly linked to user-stated goals, number of coach references to goals **Value alignment**: Number of check-ins, coach feedback/notes, goal revision frequency
Norms & Social Comparison	Compare user progress to similar peers to enhance social proof, while avoiding counter-productive pressure.	**Social Comparison:** Percentile of performance versus matched peers, competitive peer ranking and leaderboard positions, “people-like-me” benchmark view rate and dwell time, social proof banner CTR
Emotionally Resonant Feedback	Use visualizations, stories, and user-centered language to emotionally engage users with their data.	**Engagement:** Story/visualization completion rate, dwell time on narrative insights, share/bookmark rate, recall quiz accuracy **Satisfaction**: “was this helpful?” rating, pre/post sentiment shift

Behavioral outcomes serve as proximal indicators, as well as causal factors of health improvement. Sustained actions like increased activity, reduced smoking, or consistent medication use are central to evaluating whether DHIs translate digital interactions into real-world change. Example metrics include tracking steps (e.g., from devices), dietary logs, longitudinal sleep patterns, and appointment, treatment, or medication adherence. Where possible, behaviors should be objectively measured through sensor, wearable, or pharmacy refill data, rather than self-report alone.

Equally important are patient-reported outcomes (PROs), which include user-defined perceptions of sleep quality, energy, stress, and quality of life. Common measures include ratings of mood, pain, fatigue, satisfaction with progress, and perceived health status. These capture the subjective, lived experience of intervention impact and are vital for ensuring DHIs remain person-centered and aligned with individual priorities (170). When combined with passive sensor data or ecological momentary assessments (EMAs), PROs provide rich insight into both outcome and context.

Measurements must also include stratified analyses to detect disparities in engagement, adherence, and outcomes. Equity dashboards should include representation audits, engagement parity, and outcome parity across demographic, language, SES, and digital-literacy groups.

Experience and engagement metrics such as NPS, CSAT, CES, usability ratings, feature helpfulness, retention, dashboard use, streaks, completed tasks, module completion, notification responses, click-through rates, and user reviews help evaluate user satisfaction and predict long-term adherence (63, 171). Additionally, evaluating contextual fit and personalization effectiveness, including differences in literacy, cognitive load, goal achievements, changes in goal priorities, perceived fit and personalization, and DTA, helps refine targeting and content delivery (35).

#### 3.5.3 Behavioral science principles

How outcomes are communicated to users also plays a critical role in maintaining health behavior changes. Feedback that emphasizes continuous improvement (rather than binary success vs. failure outcomes) fosters persistence and a growth mindset (172, 173). Outcomes should be framed around personal relevance and goal congruence, allowing users to track their own meaningful progress (e.g., better sleep, increased confidence, walking without pain, improved mobility), rather than only abstract clinical endpoints (82, 114). Incorporating social comparisons or benchmarks (e.g., framing of “people like me”) can further enhance motivation, but these must be calibrated to avoid discouragement (174, 175). Additionally, emotionally resonant visualizations and narratives help users connect the dots between daily behaviors and long-term outcomes (176, 177).

#### 3.5.4 Other tools & tactics

Additional considerations should address key scientific and ethical challenges. These include: (a) triangulating multiple data sources to improve validity and reduce bias, for example by combining sensor data (e.g., accelerometry), self-report, and clinician-reported outcomes (177); (b) embedding contextual data (e.g., time, cost, competing life demands) in analyses, interpretation and outcome evaluation to generate actionable insights for health systems and users alike (168); and (c) acknowledging and scientifically reporting negative and null findings to enable continuous improvement and shared learning across the research field (178).

#### 3.5.5 Common challenges

The field still lacks a coherent outcomes architecture that links digital use to real-world behavior and clinical change while accounting for equity and context. Robust measurement of clinical, behavioral, and engagement outcomes remains one of the most underdeveloped areas in digital health. Product teams frequently lack the expertise or partnerships to design studies that link engagement patterns to clinically meaningful outcomes, or to collect real-world evidence via EMA, wearables, or health system data (31, 57). Passive sensing and contextual monitoring tools are rarely deployed due to technical complexity, regulatory friction, or limited interoperability with external data sources. Moreover, outcome frameworks often fail to incorporate user-defined goals or quality-of-life metrics, which are essential for personal relevance and sustained engagement (179). Evaluations are also hampered by high attrition, short follow-up windows, and limited generalizability due to unrepresentative user samples (56, 180). Without a well-resourced strategy for outcome measurement, many DHIs remain unable to demonstrate their real-world value to users, providers, or payers.

#### 3.5.6 Summary

Step 5 moves beyond convenience metrics by (a) pre-specifying the engagement → behavior →  pathway and required behavioral dose; (b) integrating multi-source data (sensors, self-report, clinician/EHR) with transparent handling of missing data; (c) including user-defined goals and quality-of-life outcomes alongside clinical markers; and (d) using pragmatic, bias-resistant designs (e.g., stepped-wedge, interrupted time series, synthetic controls) to generate decision-grade evidence in real world settings. Equity must be measured, not assumed, via subgroup reporting, fairness metrics, and audits of data coverage and dropout. Together, these practices establish outcome measurement as a pre-defined feedback loop that clarifies who benefits, by how much, and under what conditions. Practical endpoints such as the Minimum Effective Behavioral Dose (MEBD) and Time-in-Target Behavior (TTB) make the engagement-to-outcome pathway testable, comparable across studies, and actionable for both clinicians and product teams, ensuring optimization reflects user goals and values rather than platform priorities. Effective evaluation requires harmonizing digital, behavioral, and clinical outcome frameworks. Legacy metrics such as retention and click-through must be linked to behavior change and health outcomes. Measurement should also distinguish user-centered interaction metrics from person-centered, real-world impact metrics. At scale, ENGAGE aligns with continuous-learning health system principles, using real-world evidence to iteratively refine outreach, micro-engagement, and personalization over time.

### 3.6 Step 6: Expand & evolve

The final step in the ENGAGE Framework establishes a closed-loop personalization architecture in which multimodal data streams spanning clinical outcomes, behavioral and contextual signals, and real-time engagement metrics are continuously analyzed to identify what works, for whom, when, and under what conditions, and using those insights to further personalize the interventions and messaging, as well as adapting to new user needs.

This process moves DHIs from static, rule-based designs toward adaptive strategies capable of evolving dynamically over time. By integrating findings from Step 5, intervention parameters such as content, sequencing, timing, and delivery modality can be refined to maintain alignment with each user's evolving needs, capabilities, and context. Artificial intelligence (AI) and machine learning (ML) methods, particularly reinforcement learning (RL), are core mechanisms to facilitate these adaptations and enable systems to detect micro-indicators of readiness while delivering support at moments of heightened receptivity. Table 7 summarizes the core mechanisms that can be used for personalization at this stage.

**Table 7:** Core personalization techniques used in digital health with illustrative examples.

Mechanism	Key capabilities	Illustrative evidence
Conversational & Agentic AI Coaches	LLM-powered agents can be grounded in motivational interviewing techniques and deliver empathetic, personalized dialogue, negotiate goals, plan revisions, provide feedback at preferred intervals, build therapeutic alliance, triage to human support, and summarize visit or chat notes	AI chatbots can promote healthy lifestyles, treatment adherence, and substance misuse. Conversational AI-coaches have been shown to increase ambivalent smokers' readiness to quit smoking (267, 268).
Context-Aware JITAIs	Decision rules integrate real-time state (location, affect, glycaemia) and trajectory trends to trigger micro-interventions exactly when receptivity is highest	Increased effect sizes when JITAIs align with contextual cues and model (35, 186)
Data Fusion & Feature Engineering	Feature stores mapping and digital phenotyping; combining EHR, wearable, conversational and environmental data; deriving digital biomarkers (e.g., volatility of heart-rate variability as stress proxy, sleep regularity, mobility volatility)	AI-enabled remote-monitoring platforms consistently outperform rules-based systems in detecting risk escalations earlier, providing more accurate and timely alerts. This early detection enables proactive interventions, improving outcomes and reducing risk (269).
Digital-Twin Scenario Testing	Physics-informed or Bayesian Network-empowered Digital Twins can simulate treatment pathways to preview likely outcomes of alternative behavior bundles, thereby enabling individualized, data-driven dose-response optimization across multiple domains	Digital twins enable precision cardiology by tailoring treatments to individual health status and predictions of restoration pathways (270). Further work is needed to address integration and implementation challenges (188, 189)
Predictive Analytics & Risk Stratification	Calibrated risk models with monitoring, explanations, and guardrails (like contra-indication filters) can specify relevant indicators like adherence lapses or motivation dips to estimate probability of disengagement or clinical deterioration	Managers reported superior triage decisions and reduced time-to-intervention with predictive risk dashboards in chronic care programs (271)
Reinforcement-Learning Orchestration	Provides the if–then logic that links momentary state to optimal BCTs; supports reinforcement-learning reward functions focused on sustained real-world action, not clicks.	RL-guided SMS interventions significantly boosted medication adherence across chronic conditions by 14 pp vs. control using RL-guided SMS (181)
	Contextual or causal bandits can exploit message libraries and adjust to individual response curves, as well as respond to user/system constraints (e.g., fatigue, cost)	

#### 3.6.1 Reinforcement learning

Reinforcement-Learning (RL) algorithms can continuously adapt message type, timing, and channel to optimize outcomes such as medication adherence, physical-activity minutes, or other target health behaviors (181–183). By learning from ongoing feedback, RL systems move beyond static personalization toward dynamic optimization of engagement and outcomes.

#### 3.6.2 Predictive analytics

Machine-learning models increasingly integrate micro-indicators such as sentiment in text replies (e.g., “maybe later”), time-of-day usage, and passive sensor data to estimate user readiness and tailor outreach strategies (57, 184, 185). Early just-in-time adaptive intervention (JITAI) trials show that aligning message timing with perceived capability improves adherence by 20%–30% (69), and that dynamic feedback outperforms static, one-size-fits-all approaches (186, 187). Precision-health informatics, including patient-similarity networks, digital twins, and remote monitoring, further enable fine-grained adaptation and contextualization (188–190). These approaches ensure that interventions meet users where they are, balancing effective guidance with respect for readiness.

#### 3.6.3 Personalization, health equity, and digital literacy

When implemented with fairness and accessibility principles, personalization can directly reduce health inequities by tailoring content, language, modality, and cognitive load to each user's needs and capabilities. Adaptive models can adjust reading level, preferred language, notification modality (e.g., SMS vs. app), visual complexity, and timing to support individuals with lower digital literacy or limited device proficiency. Fairness-aware personalization also helps ensure that adaptive sequences do not systematically underperform for groups who have historically faced barriers to care, such as linguistic minorities, low-SES users, or those with inconsistent digital access. In this way, personalization becomes a mechanism not only for improving engagement, but for actively mitigating inequities in digital health access and outcomes.

#### 3.6.4 AI ethics

Adaptive systems must be designed with transparency, privacy, and fairness in mind (191, 192). Ethical concerns operate at both the individual level (safety, autonomy, dignity, data protection) and the societal level (fairness, accountability, surveillance, democracy, human relationships) (104, 193, 194). While numerous guidelines exist, they often lack coherence, enforcement, and stakeholder diversity (191, 195). Recommended guardrails include AI ethics checklists addressing explainability, data provenance, and bias monitoring, as well as ethical considerations embedded across the AI lifecycle (104, 196, 197). Customized AI can, paradoxically, narrow informational diversity, which warrants the use of guardrails that introduce *constructive friction* through periodic intentional insertion and exposure to alternative viewpoints, actions, small obstacles, or alternative prompts (198, 199). Constructive friction in this context refers to a design principle that supports ethical AI design by balancing efficiency with critical thinking and autonomy, while counteracting over-automation and over-personalization (i.e., over-narrowing personalization algorithms) that can create “filter bubbles” or reinforce only one type of action. AI personalization risks algorithmic bias due to unrepresentative data, language under-inclusion, and unequal sensor accuracy. Fairness must be operationalized through subgroup performance metrics, bias audits, and equity checks in RL/JITAI timing and action policies. Overall, there is consensus that current AI ethics tools need improvement in auditing and risk assessment, as they lack diverse stakeholder support and support for diverse AI development and deployment lifecycle components (106).

While reinforcement learning, digital twins, and context-aware JITAIs show promising early effects, including adaptive gains reported in small proof-of-concept trials, the current evidence base remains preliminary. Most published studies are limited by small samples, controlled settings, and narrow populations, and real-world deployment is further constrained by computational cost, data availability, privacy/security requirements, and regulatory oversight (e.g., FDA SaMD expectations and GDPR constraints). Therefore, these AI-driven approaches should be viewed as emerging capabilities rather than validated, scalable solutions, and their performance should be continuously evaluated for feasibility, generalizability, and equity.

#### 3.6.5 Evaluation & metrics

Robust evaluation frameworks are critical for monitoring AI/ML systems. Key domains include algorithmic performance (e.g., event prediction accuracy), behavioral impact (e.g., uplift analyses comparing personalized vs. static arms), engagement quality (e.g., proportion of suggestions acted upon within 24 h), clinical outcomes (e.g., biomarker changes), and equity outcomes (e.g., stratified effectiveness across demographic and digital-literacy groups, bias-detection dashboards) (183, 199, 200). Complementary user-reported metrics, such as perceptions of personalization quality, transparency, trust, and satisfaction, capture adoption potential and ensure optimization aligns with user values.

#### 3.6.6 Behavioral science principles

The behavioral science foundation of Step 6 ensures that adaptive personalization optimizes for meaningful behaviors and outcomes rather than superficial engagement. Person-action fit, as articulated in behavioral theories such as SDT and the COM-B model, informs the selection and timing of interventions to preserve autonomy, reinforce intrinsic motivation, and reduce cognitive and emotional burden. JITAI principles guide the delivery of support during periods of high receptivity, while RL models learn over time which intervention formats, sequences, and modalities yield the greatest impact. Behavioral safeguards, such as avoiding premature nudging or goal misalignment, need to be embedded to maintain trust and prevent reactance.

#### 3.6.7 Other tools & tactics

Advanced personalization workflows in this step typically involve several layers: (a) data ingestion and governance, including consented integration of EHRs, wearable sensor data, and patient-reported outcomes; (b) real-time signal interpretation via unsupervised clustering to update behavioral and clinical segmentation; (c) decision layers combining safety constraints with RL-driven optimization; (d) adaptive coach interfaces capable of multimodal delivery through human or virtual agents, with explainable rationales for recommendations; and (e) meta-learning loops that update personalization logic based on discrepancies between predicted and observed outcomes, enabling adaptation even for new users with sparse data (201–203). Early studies show that conversational agents can increase patient activation and self-management confidence by providing real-time clarification and coaching (e.g., symptom checkers, mood coaching tools). At the same time, risks such as algorithmic bias, limited explainability, and the need for human oversight remain critical considerations for safe implementation (204, 205).

#### 3.6.8 Common challenges

The practical implementation of AI- and ML-driven personalization is constrained by several persistent challenges. Many DHIs operate on siloed, static data architectures that do not support the volume, velocity, or variety of data needed to power adaptive models (50, 206, 207). Few systems are set up to ingest behavioral, contextual, and biometric data streams in real time, limiting their ability to adjust timing, tone, or content dynamically. Many product developers also lack in-house data science talent or infrastructure to train, evaluate, and monitor personalization algorithms over time. Even when models are developed, interpretability remains a barrier: black-box algorithms pose risks for user trust, clinical integration, and regulatory approval (208). Ethical concerns around bias, fairness, and consent further complicate implementation, especially in populations with limited digital literacy or historical mistrust of medical systems (209). Lastly, personalization is often framed as a static onboarding feature rather than a dynamic, closed-loop system that adapts based on longitudinal engagement and outcomes. The feasibility of real-time adaptive personalization varies widely and remains limited by data-stream availability, computational capacity, and system-level infrastructure. In many settings, personalization will occur in periodic rather than continuous updates. However, the field is still in its early stages and empirical validation of these principles remains scarce, while personalization is often confined to one dimension (201, 203). Future progress depends on integrating passive sensing, ethical AI data practices, and robust outcome modeling to realize the full promise of AI-driven digital health. Without addressing these constraints, AI-enabled precision engagement is likely to remain an aspirational objective rather than an operational reality for most digital health products.

#### 3.6.9 Summary

In Step 6, personalization operates on two levels: it adapts interventions for current users while refining the profiling and targeting of prospective ones. By integrating behavioral, clinical, and contextual profiling with predictive modeling, DHIs can improve outreach precision and ensure recruitment messages reach those most likely to benefit, thereby enhancing both initial uptake and sustained engagement through continuously updated behavioral profiles. Implemented within a rigorous behavioral science framework, AI- and ML-powered personalization can transform DHIs from reactive, episodic tools into dynamic systems that deliver timely, tailored support, maximizing adherence, clinical effectiveness, and equity. Yet these benefits require safeguards around transparency and trust, as otherwise AI risks optimizing for short-term engagement at the expense of meaningful health outcomes. By uniting behavioral science, advanced analytics, and ethical governance, Step 6 enables precision engagement to scale in a responsive, evidence-based manner capable of producing sustained health impact.

## 4 Implementation checklist

**Figure 3:**
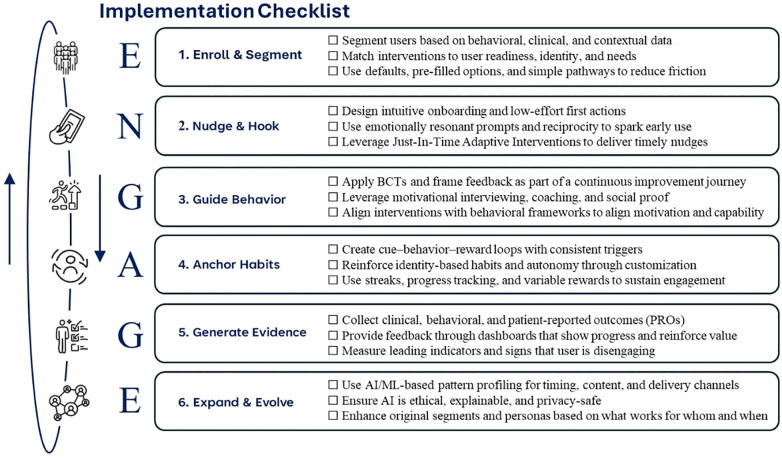
The ENGAGE framework implementation checklist.

**Figure 4:**
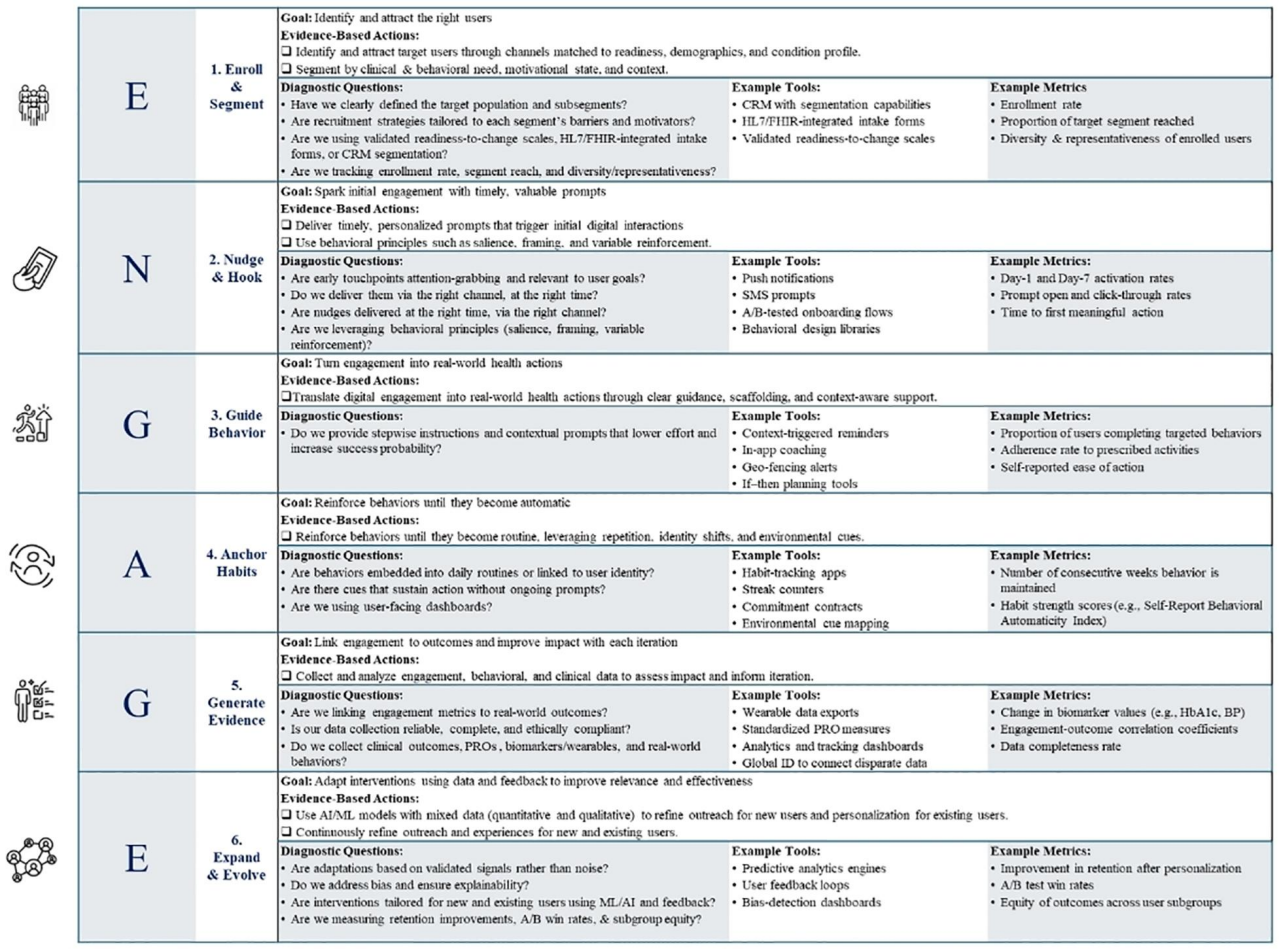
The ENGAGE framework implementation framework.

To support practitioners and researchers in translating the ENGAGE Framework from concept to execution, we developed a simple, pragmatic, six-step checklist (Figure 3). Figure 4 provides a more detailed implementation framework overview, for each step that combines (a) concrete, evidence-based actions, (b) diagnostic questions, (c) example tools, and (d) measurable indicators of success. Product teams can adopt the checklist and framework in a phase-gate approach, ensuring that no feature is launched without a clear behavioral target, theory-aligned technique, and predefined metric. Researchers can embed the same items into protocol design and reporting templates, thereby strengthening study reproducibility and cross-trial comparability.

### 4.1 How to use the checklist

The quick-reference checklist distills the behavioral science logic and theories, practical tactics, and evaluation metrics for each of the six ENGAGE steps. Innovators, product teams, and researchers can proceed column-by-column to ensure that every release, pilot, or study systematically moves users from *unawareness* to *intention* to *habit* and continuously learns “what works for whom, when, and why.”.

Teams and individuals can use this checklist for (a) sprint planning, by reviewing the different items at the start of each build-cycle to confirm which boxes will be addressed; (b) during gate reviews that require evidence of the relevant metrics before advancing to the next ENGAGE step; (c) manuscript or outcomes reporting. When describing an intervention, including a short appendix that maps intervention features and measures to the checklist items can improve reproducibility and standardization.

## 5 Discussion

The proposed six-step, cyclical ENGAGE framework synthesizes behavior-change theory, real-world context and challenges, implementation science, and recent advances in AI-enabled personalization to move DHIs beyond “time-on-app” engagement toward durable, real-world health outcomes. Comparative effectiveness evidence indicates that interventions embedding motivational techniques such as tailored feedback, personalized goal-setting, and social support consistently outperform static, one-size-fits-all programs in chronic-disease self-management (187).

### 5.1 Implications

By operationalizing these techniques in a closed-loop architecture, where leading indicator and outcomes data continuously refine segmentation, timing, and content, ENGAGE aligns with emerging precision-care models that exploit multimodal data streams (e.g., wearables, EHR integration, conversational text) to deliver “right-person, right-moment” support (190). For example, RL-guided messaging in the REINFORCE pragmatic trial uplifted medication adherence by 14%, exemplifying the clinical upside of such an adaptive approach (182). For health-system leaders, the ENGAGE Framework provides a practical roadmap for implementing value-based care by connecting micro-engagement, sustained real-world behaviors, and measurable clinical changes along a unified measurement framework. Its integrated focus on explainability and bias monitoring also aligns with current AI ethics standards set by professional bodies, ensuring that adaptive technologies remain transparent, equitable, and clinically trustworthy (105, 196, 197, 199, 210).

### 5.2 Considering ecosystem enablers for sustainable and scalable implementation

**Figure 5:**
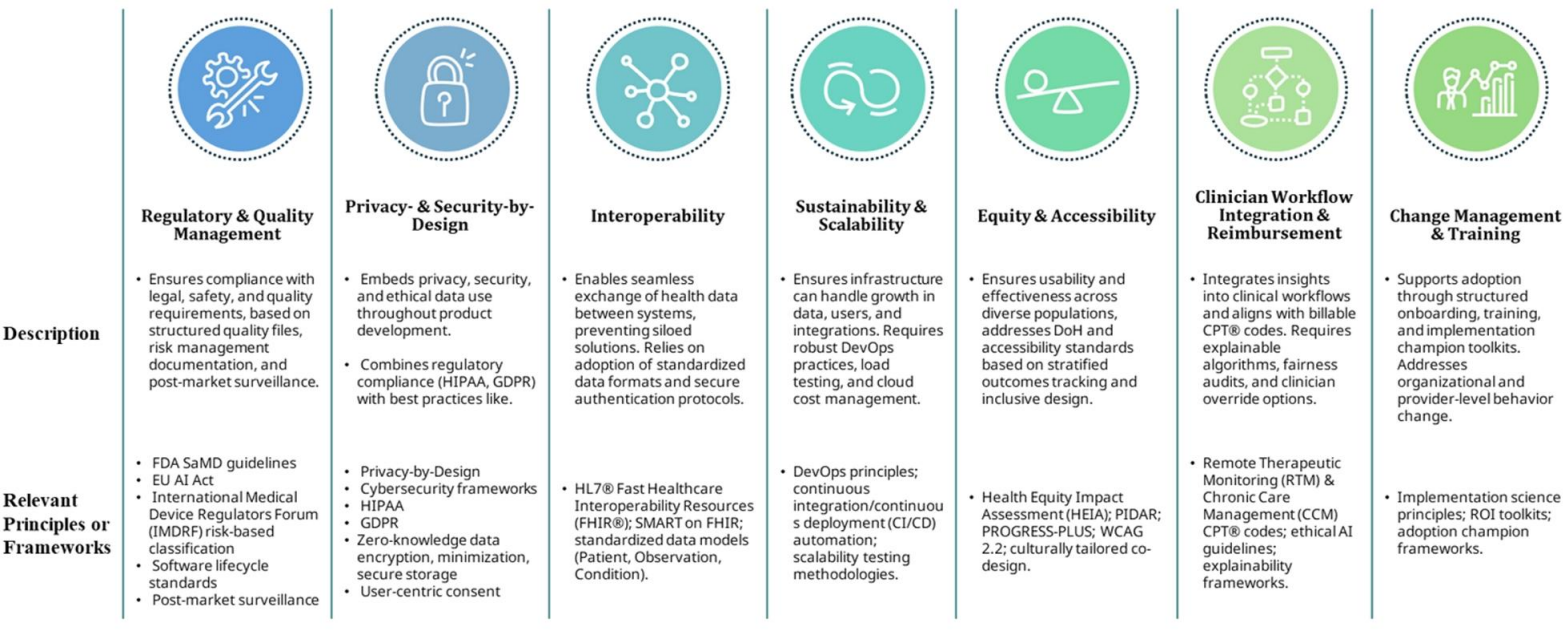
Ecosystem enablers for sustainable and scalable implementation of effective DHIs.

While the ENGAGE Framework offers a stepwise approach to driving meaningful behavior change and clinical outcomes, successful real-world implementation of DHIs also requires alignment with a broader set of cross-cutting enablers that support scalability, trustworthiness, integration, and long-term viability within complex healthcare ecosystems (211, 212). These ecosystem enablers include regulatory compliance, privacy-by-design, interoperability, equity guardrails, workflow integration, scalable operations, and structured change-management (Figure 5). Without developing strategies to overcome potential barriers in these domains, digital health products might not “survive in the wild”, even with successful user adoption, engagement, and positive clinical outcomes.

Additionally, it is important to acknowledge the tensions between multiple stakeholders that might have competing priorities, like safety for clinicians, timely results for patients, and cost efficiency for developers (38, 213). Although centered on user behavior, ENGAGE is designed to function as a boundary structure for multi-stakeholder ecosystems and can align the needs of patients, clinicians, developers, and health systems by anchoring engagement decisions in contextual fit and system-level enablers. For example, clinician safety and workflow fit are emphasized in Step3: Guide Behaviors and Step 5: Generate evidence, while patient-centered timeliness and usability are emphasized in Step 2: Nudge & Hook and Step 4: Anchor Habits, while cost and feasibility considerations are addressed in the outcomes architecture and implementation enablers. It thereby allows teams to map patient, clinician, and organizational goals onto the same engagement steps so that trade-offs, such as safety, timeliness, and cost, can be made explicit and jointly optimized.

#### 5.2.1 Regulatory and quality management compliance

First, regulatory and quality management compliance is critical. As digital health products increasingly fall under the scope of Software as a Medical Device (SaMD) classification (214), frameworks such as the U.S. FDA's SaMD guidelines, the EU AI Act, and the International Medical Device Regulators Forum (IMDRF) provide widely-adopted definitions and risk-based classification systems. They also demand rigorous documentation of risk management, software lifecycle controls, and post-market surveillance. Innovators must proactively assess regulatory classification and maintain structured quality files that meet these evolving requirements (191, 195, 214).

#### 5.2.2 Privacy- and security-by-design principles

In parallel, privacy- and security-by-design principles require a multi-layered approach, combining technical safeguards, regulatory compliance, and ethical frameworks, which all must be embedded throughout the product development lifecycle (104–106). With heightened public scrutiny of data use in healthcare, digital tools must demonstrate adherence to HIPAA, GDPR, and cybersecurity best practices such as zero-knowledge encryption, data minimization and purpose limitation, secure data storage, continuous risk assessment, and user-centric consent flows. These measures are not only legal imperatives but essential trust-builders (107, 108, 215).

#### 5.2.3 Interoperability

A third enabler is interoperability: the ability of different health technologies to talk to each other. One of the most widely adopted approaches is *HL7® Fast Healthcare Interoperability Resources (FHIR®),* a global standard for how health information is formatted and shared between systems. As FHIR becomes the norm for exchanging clinical data in the U.S. and internationally, DHIs will need to structure their data to match FHIR's format (for example, using common categories like *Patient*, *Observation*, or *Condition*) and use *SMART on FHIR* protocols to connect securely with EHR systems. Without these capabilities, digital health products risk being isolated from the broader healthcare ecosystem or rejected by health systems altogether (112, 216).

#### 5.2.4 Sustainability and scalability

For digital innovations to scale, most applications and cloud operations require a robust Development & Operations (DevOps) infrastructure. Underinvestment in backend architecture, such as insufficient data storage, lack of data integration, and inadequate infrastructure, like Global IDs (universal unique identifiers that consistently tag an individual user across different healthcare systems or programs), has been repeatedly identified as a key barrier to scaling digital health pilots (112). Common issues include unanticipated data volume, lack of national digital IDs for interoperability, lack of collaboration between developers, testers and operations teams, and technical challenges in integrating with existing health systems. These technical shortcomings often require costly redesigns or limit the ability to expand beyond the pilot phase. Additionally, load testing, continuous integration (CI) and deployment (CD) automation, cloud cost monitoring, or general product strategy renewals based on enhanced digital capacities must be part of early planning and road-mapping (217).

#### 5.2.5 Equity and accessibility

As mentioned before, equity and accessibility guardrails are essential. DHIs should stratify engagement and outcomes by factors such as race, ethnicity, primary language, rurality, and social determinants of health (SDoH). Accessible design, including WCAG 2.2 compliance, plain-language modes, and low-bandwidth alternatives, ensures broader usability, especially for underserved populations (92, 218). Multiple frameworks now guide the assessment of digital health equity, emphasizing the need to address digital determinants of health (DDoH) at individual, community, and societal levels, and the need to integrate equity considerations throughout intervention design, implementation, and evaluation (92, 99). Strategies include co-design with marginalized communities, user-friendly and culturally appropriate interfaces, infrastructure support (e.g., devices, connectivity), and digital literacy education (1, 99). Additionally, tools and frameworks like the Health Equity Impact Assessment (HEIA), PIDAR Framework, and the PROGESS-PLUS Framework provide structured checklists and recommendations that can help to improve the impact of health equity efforts (218–221).

#### 5.2.6 Clinician workflow integration and reimbursement

Clinical workflow integration plays a pivotal role in healthcare ecosystem adoption. Products that require clinicians to toggle between systems or that do not map onto billable CPT® codes (e.g., for Remote Therapeutic Monitoring, Mental Health Assessments) face major adoption barriers (222–224). DHIs must generate discrete, actionable insights that can be integrated into clinical workflows and documentation while enabling reimbursement when appropriate. As AI/ML tools are embedded into personalization engines, ethical AI governance and transparency become central for providers and healthcare organizations. Algorithms that support clinical decision-making must be explainable, regularly audited for fairness and bias, and capable of clinician override (225, 226).

#### 5.2.7 Change management

Finally, change management and training infrastructures for providers, organizations, and implementers are important for sustained adoption (49, 50, 227). Oftentimes, digital health interventions are not plug-and-play, they require human adoption and behavior change at the provider and organizational levels. Structured onboarding, role-specific training modules, and embedded “coach modes” can accelerate clinician buy-in (255–261). Toolkits for implementation champions, including ROI calculators, presentation decks, and FAQs, can further support adoption (50, 228, 229).

Together, these ecosystem factors enable the ENGAGE Framework to fully reach its potential that aligns with clinical, technical, regulatory, and equity standards. Considering them in the development process will further enhance the effectiveness of the framework and is a foundational building block to delivering real-world outcomes, earning stakeholder trust, and ensuring long-term viability at scale.

### 5.3 Limitations

The ENGAGE Framework is a theory-driven synthesis that draws on heterogeneous evidence, both based on published research and practical insights based on industry best practices, rather than being an empirically validated or tested care pathway model. A large portion of the existing research evidence comes from small or short-term studies conducted in often narrowly defined populations. The empirical validation of these findings in varied clinical settings and full generalizability across conditions and cultures remains to be tested and validated (210). Heterogeneity in engagement definitions and measurement (e.g., logins vs. affective involvement) complicates meta-analytic inference and may obscure true dose-response relationships. AI personalization layers introduce additional constraints: data completeness, model drift, and privacy requirements may limit feasibility in resource-constrained settings, while opaque algorithmic decisions can erode user trust if interpretability safeguards are absent (230). Finally, the framework presumes interoperable data infrastructure and behavioral science capacity that many organizations do not yet possess.

### 5.4 Research gaps and future directions

Standardized engagement taxonomies are still missing. Recent reviews call for harmonized, multidimensional engagement metrics that capture behavioral, cognitive, and affective facets across intervention phases (210), while consensus reporting standards akin to CONSORT-EHEALTH should be prioritized (180).

Additionally, head-to-head trials of adaptive vs. static designs are urgently needed. Few RCTs directly compare ML-driven personalization with expert-rule or generic content. Pragmatic, real-world trials, similar in scale to REINFORCE, are needed across diverse chronic conditions (182). Similarly, DTA measurements and psychometrically robust DTA scales should be validated for fully automated, human-AI hybrid, and coach-supported modalities, particularly given encouraging early results for hybrid coaching models (121, 139).

Furthermore, equity and bias surveillance are topics that should be top of mind for future research. Algorithmic performance must be audited across age, sex, ethnicity, and digital-literacy stratification to mitigate data-representation gaps. The field would benefit from more direct comparisons between rule-based vs. ML-driven personalization algorithms, especially in RCTs.

When it comes to longitudinal habit-formation trajectories, studies should link changes in habit strength to subsequent reductions in healthcare utilization and cost, enabling value-based care modelling for payers.

Lastly, human-in-the-loop optimization is still the prevailing recommendation. Emerging evidence suggests that clinician- or coach-guided recommendations that can override AI-generated decision support can improve safety and acceptance of AI recommendations (231, 232). Implementation science approaches that promote the systematic uptake of research findings and evidence-based practices into real-world healthcare and policy settings are required to integrate such “guardrails” at scale.

## 6 Conclusion

Digital health is at an inflection point: evidence shows that AI-enabled personalization can materially enhance adherence and outcomes, but only when grounded in rigorous behavioral science and evaluated with clinician-relevant endpoints (181, 203, 233). The ENGAGE Framework provides a structured, cyclical blueprint to orchestrate these elements, segmenting the right users, catalyzing micro- and macro-engagement, reinforcing habits, measuring outcomes rigorously, and adapting and personalizing interventions in real time. The framework repositions engagement as a means, not an end. By orchestrating behavioral theory, adaptive personalization, and rigorous evaluation, DHI developers can move beyond tracking clicks to transforming lives and achieving the “Goldilocks” balance of depth and duration required for long-term health impact. By embedding ethical, equitable, and theory-aligned design principles from conception through deployment, the ENGAGE Framework aspires to convert fleeting digital interactions into sustained behavior change and clinically meaningful benefits across chronic-condition trajectories.

## Data Availability

The original contributions presented in the study are included in the article/Supplementary Material, further inquiries can be directed to the corresponding author.
